# Sequence rules for a long SPOP-binding degron required for protein ubiquitylation

**DOI:** 10.1042/BCJ20253041

**Published:** 2025-05-21

**Authors:** Linda Makhlouf, Mukul Mishra, Hannah Makhlouf, Iain Manfield, Luca Busino, Elton Zeqiraj

**Affiliations:** 1 Astbury Centre for Structural Molecular Biology, School of Molecular and Cellular Biology, Faculty of Biological Sciences, University of Leeds, Leeds LS2 9JT, UK; 2 Department of Cancer Biology, Perelman School of Medicine, University of Pennsylvania, Philadelphia, Pennsylvania, USA

## Abstract

The adaptor protein, speckle-type BTB/POZ protein (SPOP), recruits substrates to the cullin-3-subclass of E3 ligase for selective protein ubiquitylation. The Myddosome protein, myeloid differentiation primary response 88 (MyD88), is ubiquitylated by the SPOP-based E3 ligase to negatively regulate immune signaling; however, the sequence rules for SPOP-mediated substrate engagement and degradation are not fully understood. Here, we show that MyD88 interacts with SPOP through a long degron that contains the established SPOP-binding consensus and an N-terminal site that we name the Q-motif. Based on the sequence similarity to MyD88, we show that additional substrates, including steroid receptor coactivator-3, SET domain-containing protein 2, and Caprin1, engage SPOP in this manner. We show that the Q-motif is a critical determinant of these interactions in mammalian cells and determine X-ray crystal structures that show the molecular basis of SPOP associations with these proteins. These studies reveal a new consensus sequence for substrate-binding to SPOP that is necessary for substrate ubiquitylation, thus expanding the sequence rules required for SPOP-mediated E3 ligase substrate recognition.

## Introduction

The post-translational ubiquitylation of substrate proteins provides a means of directing their proteasomal degradation and regulates their protein–protein interactions in a variety of cellular processes [1]. Ubiquitylation is mediated by a diversity of E3 ligase complexes, and understanding the mechanisms of substrate recruitment is key to understanding specificity and selectivity in protein degradation. The cullin (Cul)-RING ligases are multisubunit E3s that recruit substrates via their receptor and adaptor components. The Cul3-Rbx1 subclass uses a variety of BTB domain adaptors such as the speckle-type BTB/POZ protein (SPOP) [2]. SPOP recruits substrates via its N-terminal MATH domain [2], whereas a central BTB domain mediates dimerization and interactions with Cul3 [2,3]. The C-terminal BACK domain of SPOP contains an additional dimerization interface that is involved in the formation of high-order SPOP oligomers [3–5].

The SPOP MATH domain interacts with serine/threonine-rich degrons of its substrate proteins, and a five-residue SPOP-binding consensus (SBC) motif, φ-π-S-S/T-S/T (φ is a nonpolar residue, π is a polar residue, and SBC positions denoted + 1 to + 5), was initially identified in the substrates Puckered (Puc), death domain-associated protein 6 (Daxx), macroH2A, and cubitus interruptus (Ci) [2]. SBC residues interact in an extended conformation with a groove on the surface of the MATH domain [2]. The SBC has subsequently been found in additional substrates involved in diverse physiological processes [6]. SBCs are typically found in intrinsically disordered regions of the substrate, and some substrates contain several low-affinity degrons that together mediate binding to multiple MATH domains within a SPOP oligomer [2,7].

Myeloid differentiation primary response 88 (MyD88) is a component of the Myddosome complex that plays a role in the cellular response to systemic infection and tissue damage [8,9]. The Myddosome serves as a signaling relay between activated toll-like receptors (TLRs) and downstream effectors that are involved in emergency hematopoiesis, production of pro-inflammatory cytokines, and cancer development [8,10–12]. MyD88 contains an N-terminal death domain that oligomerizes and binds to interleukin-1 receptor-associated kinases, and a C-terminal toll-interleukin-1 receptor (TIR) domain that associates with TLRs [13–15]. SPOP plays a critical role in negatively regulating Myddosome signaling through the Lys^48^-linked ubiquitylation and subsequent degradation of MyD88 [16–19]. An SBC motif, ^134^VDSSV^138^, is located between the MyD88 death and TIR domains [16,18]. The +5 residue of this sequence deviates from the established SBC, and it remains unclear how this affects the SPOP-MyD88 interaction.

By evaluating the details of MyD88 interaction to SPOP, we found that MyD88 engages SPOP through a degron that is longer than the previously defined SBC, and that this is required for SPOP-mediated degradation. We identify critical residues that contribute to high-affinity interactions and show that additional substrates engage SPOP in this manner. We thereby define a new SPOP degron that contains both the established SBC and a previously uncharacterized N-terminal site that we name the Q-motif. We determine the structural basis of these interactions and show that the Q-motif interacts with an unexplored binding pocket on the SPOP MATH domain. These studies broaden our understanding of SPOP-binding degrons and will be relevant in systematic searches for new SPOP substrates and in identifying targetable degron interactions that are relevant in disease.

## Results

### The SPOP-interacting region of MyD88 is longer than the established SBC

**Figure 1:**
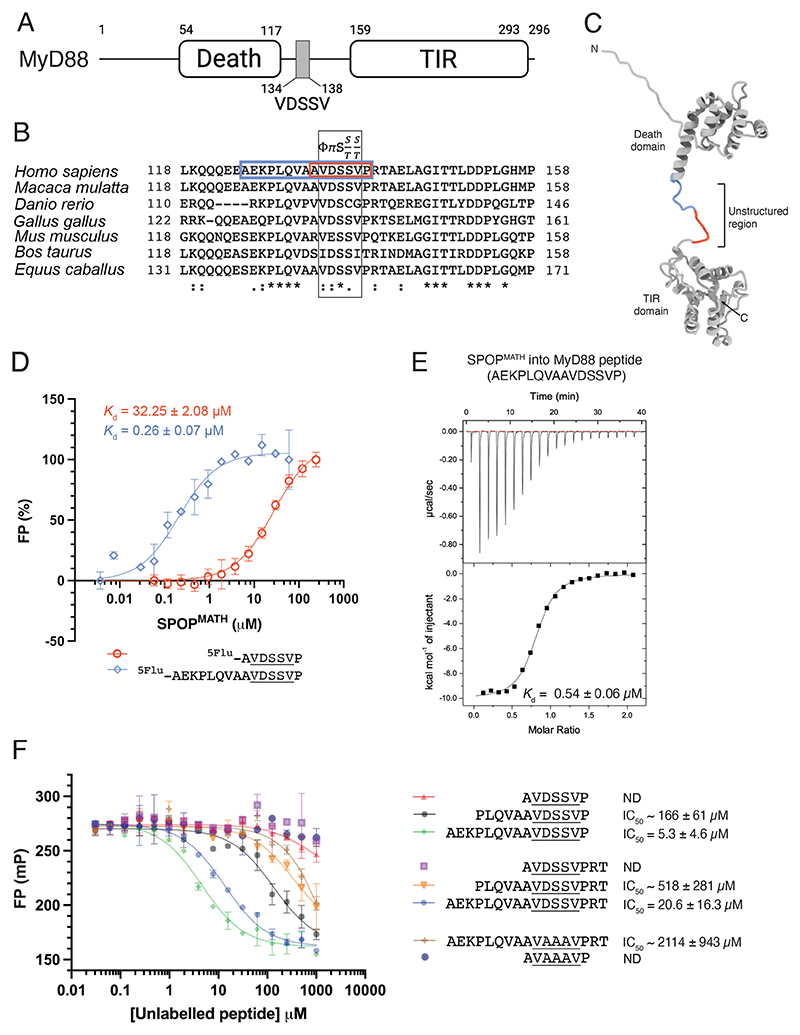
The SPOP-interacting region of MyD88 is longer than the established SBC (**A**) Domain organization of MyD88. ^134^VDSSV^138^ is the SPOP-binding consensus (SBC) motif. (**B**) Multiple-sequence alignment of MyD88. Φ-Π-S-S/T-S/T is the SBC, where Φ represents a non-polar amino acid and ∏ represents a polar amino acid. Sequences were aligned using the UniProt Clustal Omega sequence alignment tool [65]; * represents an identical residue; : represents a conserved residue;. represents a semi-conserved residue. MyD88 residues used for fluorescence polarization (FP) assays (**D**) are indicated in colored boxes. (**C**) AlphaFold2 [66–68] model of MyD88. Colors correspond to the MyD88 peptides used in FP assays (see **B** and **D**). (**D**) Binding of fluorescently (5Flu) labeled MyD88 peptides to SPOP^MATH^. SBC sequences are underlined. mP is millipolarization units. FP values were normalized to 0 and 100% polarization. Graph shows a representative experiment, and error bars are standard deviation from three (^5Flu^-AEKPLQVAAVDSSVP) or six (^5Flu^-AVDSSVP) technical replicates. Average *K*_d_ values and their standard deviation were calculated from three independent experiments. (**E**) ITC analysis for titration of SPOP^MATH^ into a MyD88 peptide. The upper panel shows representative raw ITC data, and the lower panel is the corresponding integrated heat plot. Average *K*_*d*_ values with standard deviation were calculated from three independent runs. (**F**) FP competition assay measuring the binding of a fluorescently labeled Puc peptide to SPOP^MATH^ in the presence of unlabeled competitor MyD88 peptides. Representative experiment is shown, and error bars are standard deviation from two technical replicates. Average IC_50_ values and their standard deviation were calculated from three independent experiments. Estimated IC_50_ values are denoted ~; ND is not determined. See also Supplementary Figure 1. MyD88, myeloid differentiation primary response 88; ITC, isothermal titration calorimetry; SBC, SPOP-binding consensus; SPOP, speckle-type BTB/POZ protein.

MyD88 interacts with SPOP through a sequence (^134^VDSSV^138^; MyD88 SBC) located between the death- and TIR domains (Figure 1A). Notably, the C-terminal residue of this sequence deviates from the established SBC (φ-π-S-S/T-S/T) identified in other SPOP substrates. An alignment of MyD88 protein sequences revealed a highly conserved region located immediately N-terminal to the SBC (Figure 1B), and an AlphaFold2 model predicted that this region is unstructured (Figure 1C; Supplementary Figure 1A). We hypothesized that this conserved sequence might contribute to SPOP binding and measured the interaction of MyD88 peptides with bacterially expressed SPOP protein (SPOP^MATH^; residues 28–166; Supplementary Figure 1B) using fluorescence polarization (FP) assays.

The fluorescently labeled (5Flu) MyD88 peptide, ^5Flu^-AEKPLQVAAVDSSVP, contained the conserved residues located N-terminal to the SBC (SBC underlined). This peptide bound to SPOP^MATH^ with a higher affinity than the minimal SBC peptide, ^5Flu^-AVDSSVP, with *K*_d_ values being 0.26 ± 0.07 μM and 32.25 ± 2.08 μM, respectively (Figure 1D). The unlabeled AEKPLQVAAVDSSVP peptide also bound to SPOP^MATH^ with high affinity as measured using isothermal titration calorimetry (ITC) (*K*_d_ = 0.54 ± 0.06 μM; Figure 1E; Supplementary Figure 1C). Together, these data indicate that MyD88 residues located N-terminal to the SBC are involved in its interaction with SPOP^MATH^.

To confirm the importance of the MyD88 residues located N-terminal to the SBC, we performed FP peptide competition assays using SPOP^MATH^ and a labeled peptide corresponding to the SPOP-binding sequence of the well-characterized substrate, Puc. The ^5Flu^-Puc peptide competed with a series of MyD88 peptides of varying length (Figure 1F). Consistent with previous data, the MyD88 peptide, ^125^AEKPLQVAAVDSSVP^139^, bound to SPOP^MATH^ with a higher affinity than all shorter MyD88 peptides tested (Figure 1F). An intermediate-length MyD88 peptide, ^128^PLQVAAVDSSVP^139^, also bound with a higher affinity than the minimal SBC peptide (^133^AVDSSVP^139^), suggesting that both the ^125^AEK^127^ and ^128^PLQVA^132^ segments contribute to the interaction with SPOP^MATH^. We also tested the interaction of peptides that contained additional MyD88 sequences, ^140^RT^141^, at the C-terminus and found that inclusion of these residues led to a slight reduction in binding affinity, although this difference is within the margin of error of the assay (Figure 1F). The interaction of MyD88 peptides was significantly reduced by mutation of the SBC residues, ^135^DSS^137^ to ^135^AAA^137^, consistent with previous reports [16]. These analyses suggest that MyD88 interacts with SPOP^MATH^ using a sequence that is longer than the previously established SBC. Although the canonical SBC motif is critical for binding, high-affinity interaction requires additional residues located N-terminal to this site.

### Structural basis of the SPOP^MATH^-MyD88 interaction

**Figure 2:**
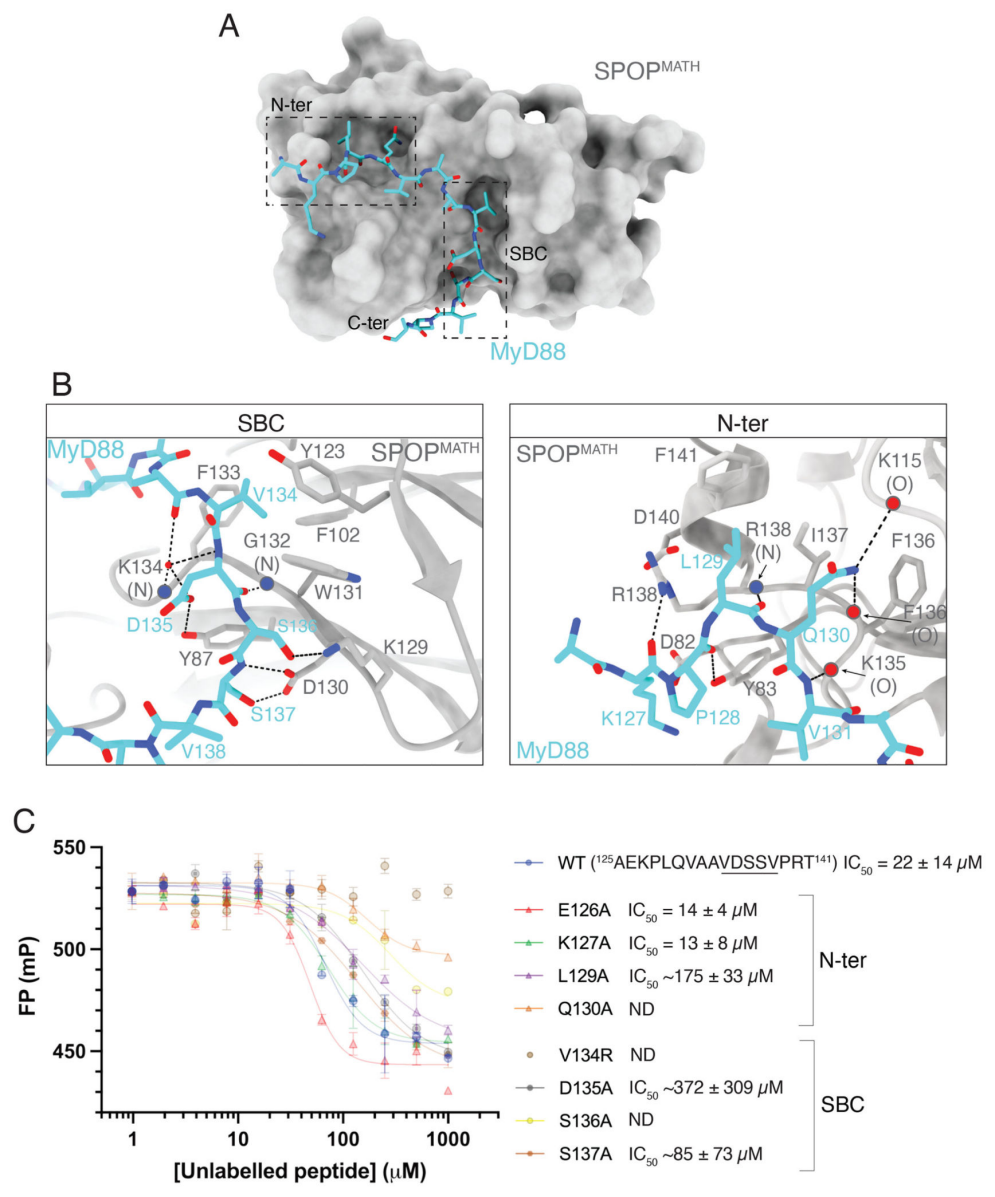
Crystal structure of SPOPMATH in complex with a MyD88 peptide (**A**) 1.9Å crystal structure of SPOP^MATH^ in complex with a MyD88 peptide (^125^AEKPLQVAAVDSSVPRT^141^). SPOP^MATH^ is displayed as a solvent-accessible surface in gray, and the MyD88 peptide is shown in stick representation in cyan. Hatched boxes denote regions of the MyD88 peptide. (**B**) Detailed view of interactions between SPOP^MATH^ and MyD88. Hydrogen bonds are shown as black dashed lines. (**C**) Fluorescence polarization (FP) competition assay measuring the binding of a fluorescently labeled Puc peptide to SPOP^MATH^ in the presence of unlabeled competitor MyD88 peptides containing single amino acid substitutions. mP is millipolarization units. Representative experiment is shown, and error bars are standard deviation from two technical replicates. Average IC_50_ values and their standard deviation were calculated from three independent experiments. Estimated IC_50_ values are denoted ~; ND is not determined. See also Supplementary Figure S2. N-ter, N-terminus of MyD88 peptide; C-ter, C-terminus of MyD88; SBC, SPOP-binding consensus.

To understand the structural basis of the MyD88-SPOP interaction, we solved a 1.9 Å crystal structure of SPOP^MATH^ in complex with a MyD88 peptide, ^125^AEKPLQVAAVDSSVPRT^141^ (Figure 2A and Supplementary Figure 2A, Supplementary Table 1). In total, 15 residues of the 17-mer peptide were well resolved, with only Ala^125^ and Thr^141^ missing, most likely due to flexibility of these terminal regions. The MyD88 peptide binds in a groove on the surface of SPOP^MATH^ (Figure 2A), making extensive contacts and burying ~827 Å^2^ of surface area. The SBC residues interact at an edge β-strand in a manner observed in other SPOP substrates [2,20–26], whereas the interactions of residues located N- and C-terminal to the SBC are unique to MyD88.

The first four residues of the MyD88 SBC (^134^VDSS^137^) match the defined SBC consensus (φ-π-S-S/T-S/T), and their interactions with SPOP^MATH^ (Figure 2B) resemble those of other substrates. The side chain of MyD88 Val^134^ is buried in a hydrophobic pocket that is contributed by SPOP residues Phe^102^, Tyr^123^, Trp^131^, and Phe^133^, whereas the side chains of MyD88 residues ^135^DSS^137^ are involved in hydrogen bonding with the side chains of SPOP residues Tyr^87^, Lys^129^, and Asp^130^. MyD88 residues ^135^DSS^137^ also form main-chain- and water-mediated hydrogen bonds with SPOP^MATH^ (Figure 2B and Supplementary Figure 2B). The +5 residue of the MyD88 SBC (Val^138^) differs from the defined consensus. In other SPOP substrates, this SBC residue usually has a polar side chain that forms hydrogen bonds with SPOP Lys^129^ and Asp^130^. MyD88 Val^138^ cannot make these contacts, and instead, the backbone NH group of MyD88 Arg^140^ forms a hydrogen bond with the side chain of SPOP Asp^77^, thereby directing the C-terminus of the MyD88 peptide in a different direction from that observed in other SPOP substrates (Supplementary Figure 2C). Of note, the Cα B-factors of the C-terminal MyD88 residues, ^138^VPR^140^, were relatively high (Supplementary Figure 2D), and these residues were not modeled in a crystal structure of SPOP^MATH^ in complex with a longer MyD88 peptide (^128^PLQVAAVDSSVPRTAELAG^146^; Supplementary Figures 2E and 5A, 5B). This suggests that the residues located C-terminal to the SBC are highly flexible, as observed in structures of other SPOP substrates [2,23,24,26].

The N-terminal residues of the MyD88 peptide (^127^KPLQV^131^) form both hydrophobic- and hydrogen-bond interactions within the substrate-binding groove (Figure 2B). The side chains of MyD88 Leu^129^ and Gln^130^ sit in a hydrophobic pocket that is contributed by SPOP residues Phe^136^, Ile^137^, and Phe^141^, with the side chain of Gln^130^ also forming hydrogen bonds with the backbone carbonyl groups of SPOP Lys^115^ and Phe^136^ (Figure 2B and Supplementary Figure 2F). This binding mode is stabilized by backbone–backbone interactions between MyD88 Leu^129^ and SPOP Arg^138^ and between MyD88 Val^131^ and SPOP Lys^135^ (Figure 2B). The side chain of SPOP Tyr^83^ is involved in anchoring the peptide in this region, where it forms a hydrogen bond with the backbone carbonyl group of MyD88 Pro^128^ and contributes hydrophobic interactions with MyD88 Pro^128^ and Val^131^ (Figure 2B). At the end of the substrate-binding groove, the backbone carbonyl group of MyD88 Lys^127^ forms a hydrogen bond with the side chain of SPOP Arg^138^ (Figure 2B). Formation of this hydrogen bond involves movement of the SPOP Arg^138^ side chain relative to the unliganded state of SPOP^MATH^ (PDB ID: 7KPI [21]); this is the only apparent change in side chain positioning observed upon complex formation. MyD88 residues ^131^VAA^133^ are located in the central region of the MyD88 peptide and form a network of water-mediated contacts with SPOP^MATH^ (Supplementary Figure 2G).

### High-affinity interaction of MyD88 with SPOP requires residues N-terminal to the SBC

To examine the significance of the interactions observed in the crystal structure, we used FP competition assays to test the binding of MyD88 peptides containing single amino acid substitutions of interacting residues (Figure 2C). As reported for other SPOP substrates [2,16,18,23,26–39], substitutions along the SBC residues ^134^VDSS^137^ impaired the binding to SPOP^MATH^, likely due to disruption of the hydrophobic- and hydrogen-bond interactions observed in the crystal structure (Figure 2B and 2C). Also, in common with other substrates, mutation of the first (Val^134^) and third (Ser^136^) SBC positions had the greatest effect (Figure 2C). Binding was also impaired by mutation of residues located N-terminal to the SBC, with the effect of the L129A and Q130A mutations being of similar magnitude to some of the SBC mutations (Figure 2C). Mutation of these residues would likely disrupt the interaction of MyD88 with the hydrophobic pocket of SPOP^MATH^ (Phe^136^, Ile^137^, and Phe^141^), and mutation of MyD88 Gln^130^ would also disrupt hydrogen-bond interactions with main chain atoms of SPOP Lys^115^ and Phe^136^ (Figure 2B). Importantly, these mutations led to impaired binding in the context of 17-mer MyD88 peptides that contained a wildtype SBC. This suggests that MyD88 residues, ^129^LQ^130^, act as a second site of interaction that, together with the SBC, is required for high-affinity binding to SPOP^MATH^.

### A long degron sequence is necessary for MyD88 degradation

**Figure 3:**
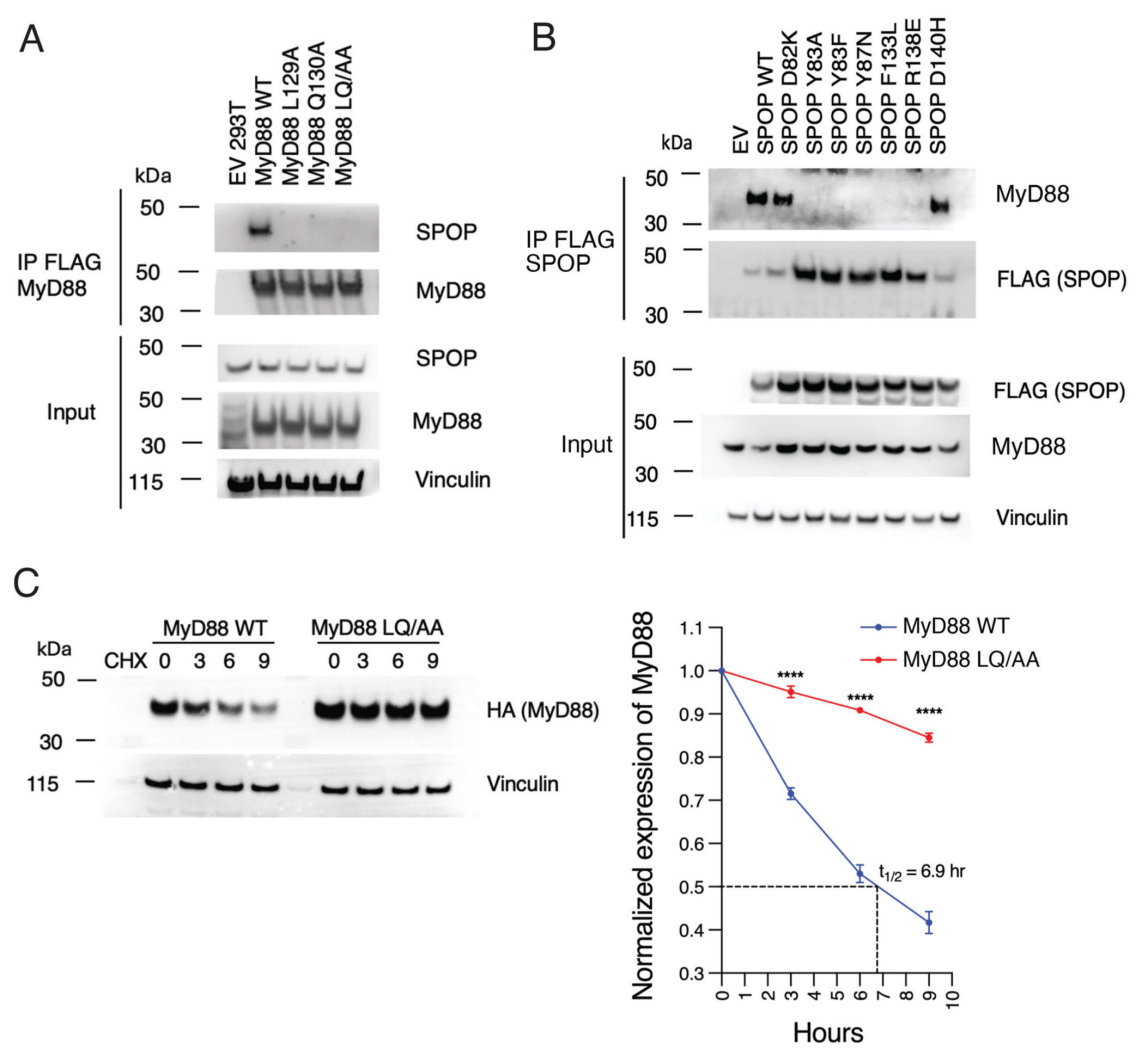
Mutational analysis of the SPOP-MyD88 interaction in cells (**A**) Immunoblot analysis for the indicated proteins from the whole cell lysate and FLAG-immunoprecipitated (FLAG-IP) samples of HEK293T cells overexpressing the indicated FLAG-tagged MyD88 mutants. The cells were treated with MLN4924 for 6 hours before harvesting. EV is empty vector. (**B**) Same as in (**A**), except that the indicated SPOP mutants were utilized. The reduction in SPOP levels in the IP FLAG (SPOP) blot is likely due the FLAG epitope being obscured upon MyD88 binding. (**C**) (Left) Immunoblot analysis for the indicated proteins in BJAB cells infected with retroviruses encoding MyD88 (WT) or MyD88 (^129^LQ^130^/AA) mutant. Cells treated with cycloheximide (CHX) for the indicated time points. (Right) quantification of MyD88 immunoblots. Relative intensity was plotted over time (mean ± SD; *n* = 3 independent experiments; two-way ANOVA; **** indicates P ≤ 0.0001). MyD88, myeloid differentiation primary response 88; SBC, SPOP-binding consensus; SPOP, speckle-type BTB/POZ protein.

We next confirmed the importance of MyD88 residues ^129^LQ^130^ in the interaction with SPOP^MATH^ in cells. The interaction between full-length SPOP and MyD88 was analyzed by co-immunoprecipitation of transiently transfected MyD88 and endogenous SPOP in HEK293T cells. Consistent with the FP assays, the cellular interaction was abolished by both single and double mutation of MyD88 residues ^129^LQ^130^ into alanine (Figure 3A). We correspondingly tested the effect of mutating SPOP residues (Tyr^87^ and Phe^133^) predicted to interact with the MyD88 SBC, and SPOP residues (Tyr^83^, Asp^82^, and Arg^138^) predicted to interact with the N-terminal portion of the MyD88 SPOP-binding sequence (Figure 3B). We also analyzed a SPOP cancer-associated mutation, D140H [18] that is located near the substrate-binding groove, close to the N-terminal MyD88 residues. As predicted, the interaction with MyD88 was abolished by mutation of the SBC-interacting SPOP residues (Y87N and F133L). The interaction was also abolished by the SPOP Y83A, Y83F, and R138E mutations, consistent with a role for these residues in anchoring the ^129^LQ^130^ N-terminal portion of the MyD88 SPOP-binding sequence. Mutation of SPOP Asp^82^ did not abolish the interaction, most likely because this residue is only involved in side chain contacts with the highly flexible MyD88 Lys^127^ at the end of the interaction site. Additionally, mutation of SPOP Asp^140^ (D140H) did not affect its interaction with MyD88 in this assay.

To assess the functional effect of degron ^129^LQ^130^ mutations, we determined the stability of MyD88 protein using cycloheximide chase assays in BJAB cells stably expressing MyD88 wild-type or ^129^LQ^130^/AA mutant (Figure 3C). MyD88 half-life was prolonged upon mutation of ^129^LQ^130^ residues, consistent with their role in interacting with SPOP, and consistent with previous studies showing that MyD88 degradation is regulated by SPOP-mediated ubiquitylation [16,17]. Together, these data confirm that MyD88 residues ^129^LQ^130^ are critical for its binding to SPOP, thereby mediating subsequent ubiquitylation and degradation.

### SPOP substrate degrons – importance of both the SBC and an N-terminal Q-motif

Individual substrate degrons interact with SPOP with a wide range of binding affinities [2,7,24,29]. We reasoned that the strength of degron interaction might depend on the sequence of its SBC and in some cases, also of residues located N-terminal to this site.

**Figure 4:**
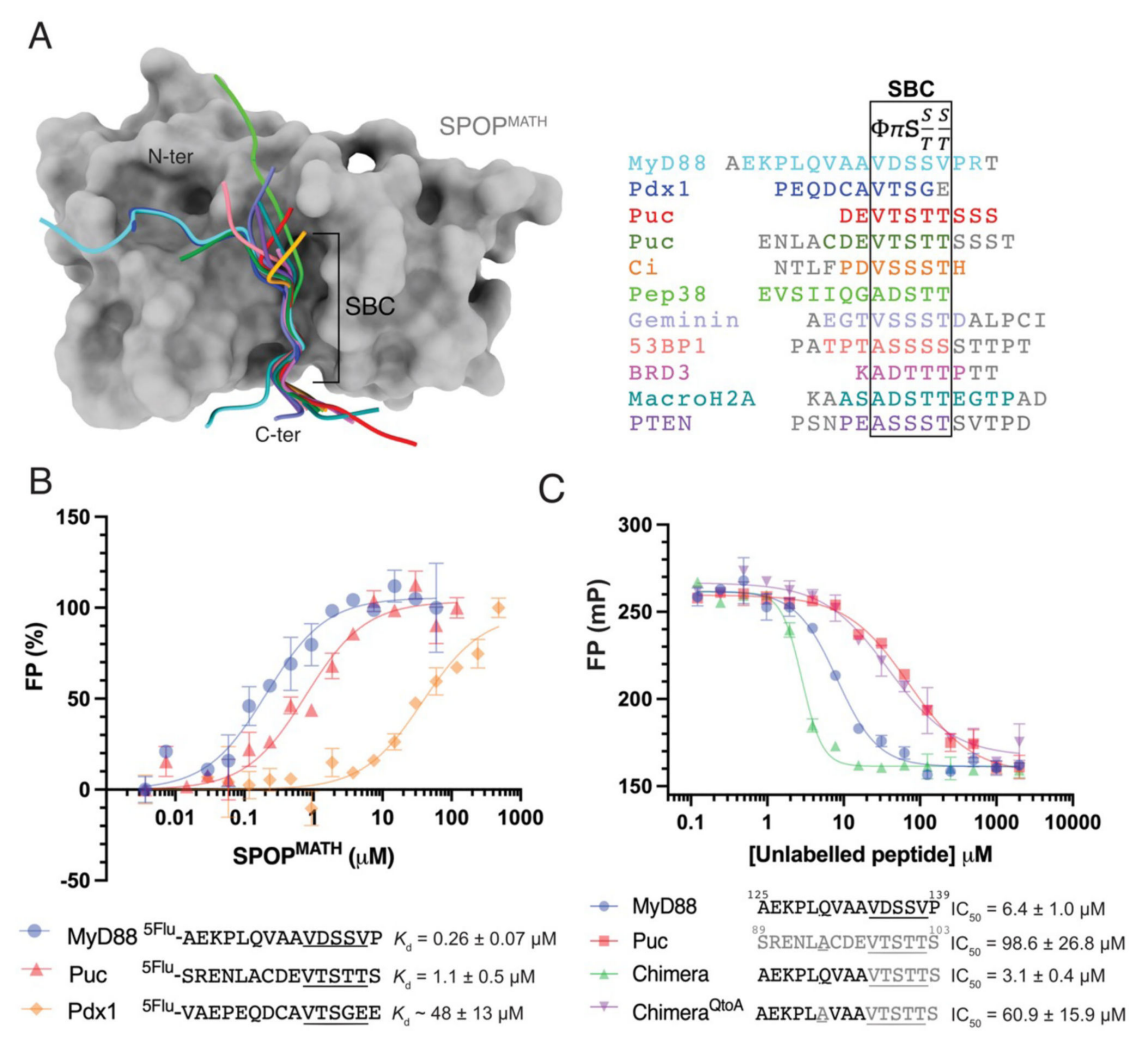
SPOP substrate degrons – importance of both the SBC and an N-terminal Q-motif (**A**) Comparison of independent structures of SPOP^MATH^ in complex with various substrate peptides. Superposition was by structural alignment of the MATH domains. SPOP^MATH^ from the complex with MyD88 is displayed as a solvent-accessible surface in gray; substrate peptides are shown as cartoons in colors corresponding to the sequences on the right. Residues depicted in gray were present in the peptides used for crystallization but were not built into the models. Boxed residues are the SPOP-binding consensus (SBC; Φ-Π-S-S/T-S/T) sequence, where Φ represents a non-polar amino acid and Π represents a polar amino acid. Pdx1, PDB ID: 6F8F [20]; Puc^SBC1_pep1^, PDB ID: 3IVV [2]; Puc^SBC1_pep2^, PDB ID: 3HQL [2]; Ci^SBC2^, PDB ID: 3HQM [2]; Pep38, PDB ID: 7D3D [22]; Geminin, PDB ID: 7KLZ [23]; 53BP1, PDB ID: 7LIN [24]; BRD3, PDB ID: 6I7A [25]; MacroH2A^SBC_pep1^ PDB ID: 3HQH [2]; PTEN, PDB ID: 4O1V [26]; MyD88, this study. (**B**) Binding of fluorescently labeled substrate peptides to SPOP^MATH^. SBC sequences are underlined. Fluorescence polarization (FP) values were normalized to 0 and 100% polarization. Graph shows representative experiment, and error bars are standard deviation from two technical replicates (MyD88 data shown are the same experiment as with Figure 1D). Average *K*_d_ values and their standard deviation were calculated from three independent experiments. (**C**) FP competition assay measuring the binding of a fluorescently labeled Puc peptide to SPOP^MATH^ in the presence of competitor unlabeled MyD88, Puc or chimeric peptides. mP is millipolarization units. MyD88 residues are shown in black and Puc residues in gray. SBC sequences and the N-terminal glutamine residue are underlined. Graph shows representative experiment, and error bars are standard deviation from two technical replicates. Average IC_50_ values and their standard deviation were calculated from three independent experiments. See also Supplementary Figure 3. MyD88, myeloid differentiation primary response 88; SBC, SPOP-binding consensus; SPOP, speckle-type BTB/POZ protein.

Pancreas/duodenum homeobox protein 1 (Pdx1) is a SPOP substrate that interacts with SPOP using residues N-terminal to its SBC [20,21]. Comparison of independent structures of SPOP^MATH^ in complex with various substrate peptides (Figure 4A) showed that the MyD88 and Pdx1 peptides superimpose well in their N-terminal region (RMSD = 0.663 between 11 matching Cα atoms). Notably, Pdx1 Gln^225^ is oriented identically to MyD88 Gln^130^ in a hydrophobic pocket of SPOP^MATH^ (SPOP Phe^136^, Ile^137^, and Phe^141^), with its side chain forming identical hydrogen bonds with the backbone carbonyl groups of SPOP Lys^115^ and Phe^136^ (Supplementary Figure 4). The main chain atoms of Pdx1 Pro^223^, Glu^224^, and Asp^226^ also form identical interactions to those of MyD88 Pro^128^, Leu^129^, and Val^131^. These residues interact with SPOP Tyr^83^, Arg^138^, and Lys^135^, respectively, thereby anchoring the N-termini of the MyD88 and Pdx1 peptides in the same orientation within the substrate-binding groove. No other substrate peptides have been reported to interact with this region of SPOP^MATH^. Although some crystallization conditions contained long peptides that might potentially extend this far, the relevant N-terminal residues were not modeled into the electron density of the high-resolution crystal structures (Figure 4A).

The SPOP substrate, Puc, contains an SBC (Puc^SBC1^) with the highest reported binding affinity to SPOP^MATH^. We used an FP assay to compare the binding affinity of 15-mer peptides containing the SPOP-binding regions of Puc (Puc^SBC1^), Pdx1, and MyD88. Each peptide had identical boundaries as aligned via their SBCs, and they contained N-terminal residues corresponding to MyD88 ^129^LQ^130^ (Figure 4A, right panel). The *K*_d_s of these interactions were 1.1 ± 0.07 μM (Puc), ~ 48 ± 13 μM (Pdx1), and 0.26 ± 0.07 μM (MyD88). The *K*_d_ values of the Puc and Pdx1 interactions are similar to those reported using a variety of techniques for peptides with slightly different boundaries [2,20,40]. These results suggest that the SPOP-binding region of MyD88 interacts with a higher affinity than all previously characterized SPOP degrons.

Notably, the SBCs of both MyD88 (^134^VDSSV^138^) and Pdx1 (^229^VTSGE^233^) deviate from the consensus sequence (φ-π-S-S/T-S/T) at the C-terminus. We, therefore, reasoned that the binding of peptides containing ‘non-optimal’ SBCs might be dependent on interactions mediated by residues N-terminal to this site. Conversely, the high-affinity binding of the Puc peptide, despite not interacting via residues N-terminal to the SBC, might reflect the good match of its SBC (^98^VTSTT^102^) to the consensus.

To test this hypothesis, we used an FP competition assay to measure the binding of a chimeric peptide, AEKPLQVAAVTSTTS, that comprised the N-terminal region of the MyD88 SPOP-binding sequence (AEKPLQVAA) fused to the Puc SBC (VTSTTS) (Figure 4C). The chimeric peptide bound to SPOP^MATH^ with a higher affinity than either the MyD88 or Puc peptides. Notably, the natural Puc sequence has an alanine residue (Puc Ala^94^) at the position corresponding to MyD88 Gln^130^. Given the importance of this glutamine residue in the SPOP^MATH^-MyD88 interaction (Figure 2C), we mutated it to alanine in the context of the chimeric MyD88-Puc peptide. This mutation led to a reduction in binding of the chimeric peptide, resulting in an IC_50_ value similar to that of the natural Puc peptide (Figure 4C). Together, these results suggest that the SBC of Puc binds to SPOP^MATH^ with a higher affinity than the SBC of MyD88. They also show that the N-terminal glutamine residue is required for the high-affinity interaction of the chimeric MyD88-Puc peptide, consistent with the importance of Gln^130^ in the MyD88-SPOP interaction. The strength of degron interaction with SPOP^MATH^ is therefore determined by the sequence of its SBC and, in some cases, also of residues N-terminal to this location. To reflect the importance of MyD88 Gln^130^ residue, we refer to this region as the Q-motif.

### Additional substrates interact with SPOP^MATH^ via a Q-motif

**Figure 5:**
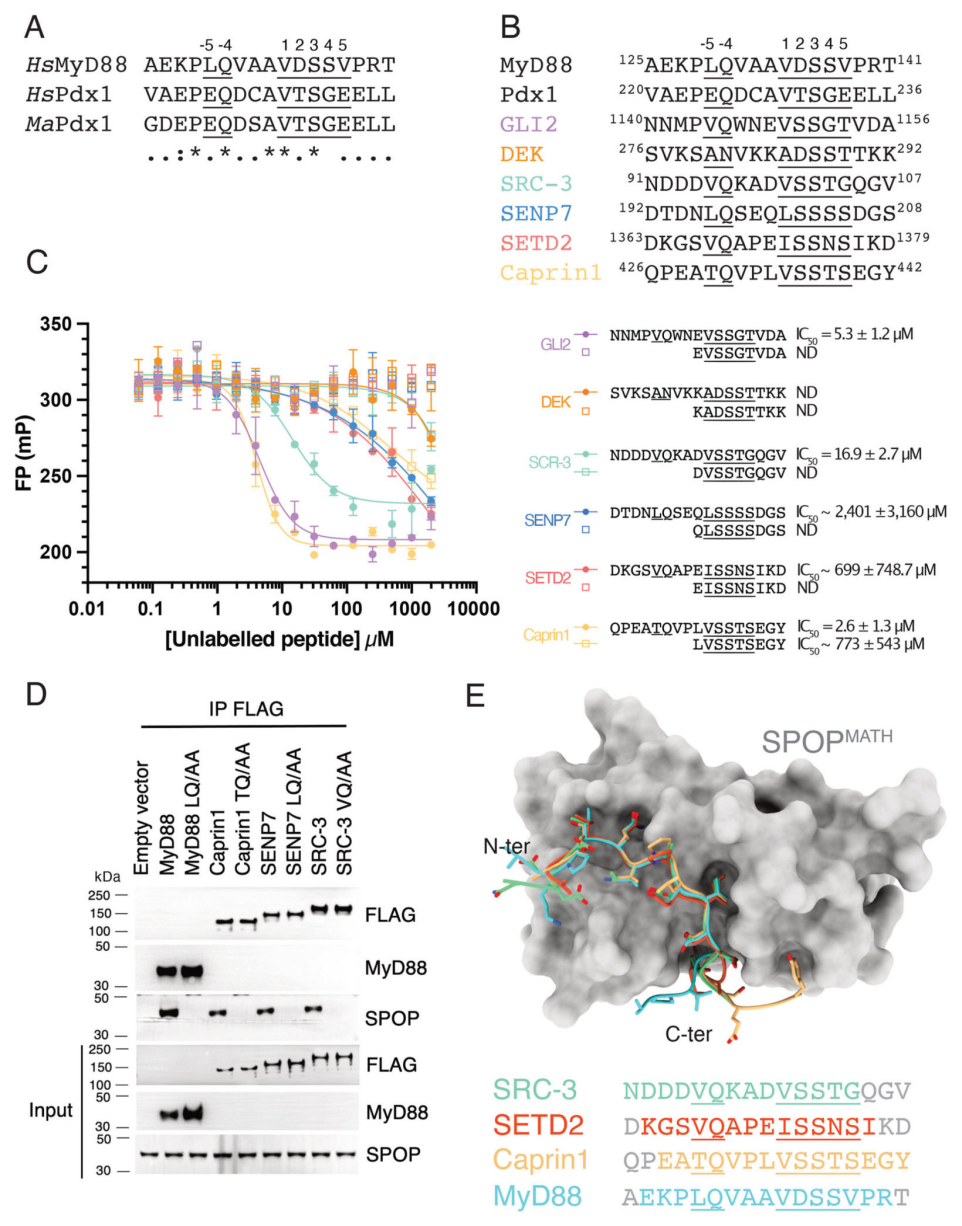
Additional substrates interact with SPOPMATH via a Q-motif (**A**) Sequence alignment of the SPOP-binding region of MyD88 and Pdx1. *Hs* is *Homo sapiens*; *Ma* is *Mesocricetus auratus* (Golden hamster). SBC sequences (1 to 5) and the Q-motif (−5 to −4) are numbered. * represents an identical residue; : represents a conserved residue;. represents a semi-conserved residue. (**B**) Sequences of known SPOP substrates shown to have similarity with the expanded SPOP-binding search motif. (**C**) Fluorescence polarization (FP) competition assay measuring the binding of fluorescently labeled Puc peptide to SPOP^MATH^ in the presence of unlabeled competitor 17-mer and 9-mer substrate peptides. mP is millipolarization units. Representative experiment is shown, and error bars are standard deviation from two technical replicates. Average IC_50_ values and their standard deviation were calculated from three independent experiments. (**D**) Immunoblot analysis for the indicated proteins from the whole cell lysate and FLAG-immunoprecipitated (FLAG-IP) samples of HEK293T cells overexpressing the indicated FLAG-tagged proteins. The cells were treated with MLN4924 for 6 hours before harvesting. (**E)** Comparison of independent crystal structures of SPOP^MATH^ in complex with substrate peptides from MyD88 (cyan), SRC-3 (green), SETD2 (red), and Caprin1 (yellow). Structures were superposed by structural alignment of the SPOP^MATH^ domains. SPOP^MATH^ from the complex with Caprin1 is displayed as a solvent-accessible surface in gray, and substrate peptides are displayed with backbones as cartoons and side chains as sticks. Peptide sequences used for crystallization are shown on the right; residues depicted in gray were not built into the models. SBC sequences and the Q-motif are underlined. See also Supplementary Figure 4. MyD88, myeloid differentiation primary response 88; SBC, SPOP-binding consensus; SPOP, speckle-type BTB/POZ protein.

We next determined whether other SPOP substrates contain a Q-motif within their degron. We generated a 17-residue search motif based on the sequences of MyD88 and Pdx1, and on the structures of SPOP^MATH^ in complex with the corresponding peptides (Figure 5A and Supplementary Figure 4A). At each residue position, we used the crystal structures to reason which other amino acids could potentially interact with SPOP^MATH^, with particular focus on the positions corresponding to the MyD88 ^129^LQ^130^ site. Consensus positions were numbered relative to the established SBC, with the first SBC position being denoted ‘+1’ and the position corresponding to MyD88 Gln^130^ being ‘−4’. The SBC positions +4 and +5 were, thus, relaxed to allow any amino acid, and the Q-motif positions −5 and −4 contained the allowed amino acids (LVIEDQN) and (QNE), respectively (Supplementary Figure 4A). Aside from the Q-motif and the first three SBC residues, all amino acids were allowed at the other positions of the search motif, which was then manually compared with the amino acid sequences of known SPOP substrates (Supplementary Table 2). We found that the search motif is present in six additional known SPOP substrates – GLI family zinc finger 2 (GLI2) [41], DEK [30], steroid receptor coactivator-3 (SRC-3; also known as nuclear receptor coactivator 3 (NCoA-3)) [42], sentrin-specific protease 7 (SENP7) [33], SET domain-containing protein 2 (SETD2) [34], and Caprin1 [43] (Figure 5B). We then measured the interaction of substrate peptides with SPOP^MATH^ using FP competition assays (Figure 5C). We tested the binding of both 17-mer peptides that contained residues N-terminal to the SBC and of 9-mer peptides that lacked this region. Overall, the longer peptides bound to SPOP^MATH^ with a higher affinity than shorter peptides. The 17-mer peptides of Caprin1, GLI2, and SRC-3 bound with the highest affinity, whereas the DEK peptides had the lowest-affinity interactions, with little difference between the 17-mer and 9-mer peptides. Of note, the DEK sequence has an asparagine residue at the −4 position of the binding consensus. The relatively weak interactions of the DEK peptides might therefore be consistent with a requirement for a glutamine residue at this site, as found in MyD88 and Pdx1.

In cells, we confirmed the interaction of endogenous SPOP to overexpressed Caprin1, SENP7, and SRC-3 by coimmunoprecipitation in HEK293T cells (Figure 5D). Similar to MyD88, mutation of the Q-motif abolished the interactions, consistent with the finding that the Q-motif is necessary for interaction of SPOP to these proteins.

We crystallized SPOP^MATH^ in complex with the 17-mer substrate peptides and obtained high-resolution structures of SPOP^MATH^ in complex with SRC-3, SETD2, and Caprin1. The SRC-3, SETD2, and Caprin1 peptides all interact at the SPOP^MATH^ substrate-binding groove with the same overall backbone conformation as MyD88 (Figure 5E; Supplementary Figure 4B, Supplementary Table 3 and Supplementary Figure 5C-H). In the N-terminal region, the backbone atoms of peptide positions −3,–5, and −6 make identical hydrogen-bond contacts with main chain atoms of SPOP Lys^135^ and Arg^138^ and with the side chain of SPOP Tyr^83^, respectively (Supplementary Figure 4B). At the −4 position, the SRC-3, SETD2, and Caprin1 peptides all contain a glutamine residue corresponding to MyD88 Gln^130^, and the side chain of this residue makes identical contacts with the backbone carbonyl groups of SPOP Lys^115^ and Tyr^136^. The SPOP^MATH^ hydrophobic pocket (SPOP Phe^136^, Ile^137^, and Phe^141^) encompasses the side chains of peptide positions −4 and −5. The SBC regions of the SRC-3, SETD2, and Caprin1 peptides also interact with SPOP^MATH^ as reported in other substrates, with the exception of the SETD2 and SRC-3 residues that deviate from the consensus at the +4 and +5 positions, respectively (Figure 5E). The C-terminal residues of the Caprin1 peptide (Caprin1 ^440^EGY^442^) follow the same path as the C-terminus of a macroH2A peptide (PDB ID: 3IVB; macroH2A ^175^EGT^177^ [2]; Supplementary Figure 4C), with the side chains of Caprin1 Tyr^442^ and macroH2A Thr^177^ both occupying a small pocket contributed by the side chains of SPOP residues Tyr^123^, Arg^124^, Val^126^, Lys^129^, and Trp^131^.

The established SBC, φ-π-S-S/T-S/T, was initially defined in the SPOP substrates Puc, macroH2A, Daxx, and Ci [2], and we confirmed this consensus using sequences from a wider range of recently identified substrates (Supplementary Figure 4D and Supplementary Table 2). We used the SPOP-binding sequences of MyD88, Pdx1, GLI2, SRC-3, SENP7, SETD2, and Caprin1 to generate an expanded SPOP degron consensus sequence, λ-Q-X-X-X-φ-π-S-X-X (X is any amino acid, λ is a medium or large amino acid) (Figure 4E), that contains both the SBC and Q-motif.

## Discussion

SPOP terminates immune signaling by recruiting the Myddosome component, MyD88, for ubiquitylation. We show that the SPOP-binding sequence of MyD88 interacts with an affinity (*K*_d_ of ~260 nM) that is greater than that of previously characterized degrons. The SPOP-binding degron of MyD88 contains two sites that are critical for interaction – the established SBC (MyD88 residues ^134^VDSSV^138^) and a previously uncharacterized sequence (MyD88 residues ^129^LQ^130^) located N-terminal to this region. We name this second site the Q-motif and identify this motif in other known SPOP client proteins that are substrates for ubiquitylation (GLI2, SRC-3, SENP7, SETD2, and Caprin1). The ‘long’ degrons of these substrates bind in a common overall conformation within the substrate-binding groove of the SPOP MATH domain, with the Q-motif sitting in a hydrophobic pocket of SPOP^MATH^, and the glutamine residue making side chain contacts with the SPOP backbone. This work has, therefore, revealed a new feature of SPOP degrons and highlighted the importance of a previously unexplored binding pocket on the SPOP MATH domain. Our work also highlights an additional SPOP binding pocket C-terminal of the SBC, as shown by macroH2A [2] and Caprin1 binding (Figure 5 and Supplementary Figure 4). An artificial substrate peptide (Pep38) showed additional SPOP-binding N-terminal to the SBC through an extended B-sheet interaction [22]. Additionally, inclusion of amino acids N-terminal to the SBC_1 of ERG increased binding to SPOP [44]. No structures exist of this interaction, but the sequence of ERG does not contain a Q-motif and structural predictions of ERG peptides and SPOP^MATH^ showed the ERG peptide forming additional contacts with SPOP, but no binding to the Q-motif pocket. These examples illustrate the versatility of SPOP^MATH^ to selectively bind a diverse set of degron sequences.

The degrons of SPOP client proteins have a wide range of binding affinities, and many contain several low-affinity degrons that together mediate the multivalent binding to a high-order SPOP oligomer. Although MyD88 contains a well-matched SBC (^14^VSSTS^18^) in an unstructured region of its N-terminus, mutational analysis suggested that this region is not important for SPOP binding or in ubiquitylation [18]. Therefore, it is likely that MyD88 interacts with SPOP mainly via the degron described in this study. The positioning of degrons within disordered regions will generally enable both accessibility for interaction and flexibility for the ubiquitylation of a suitably positioned lysine. However, it is relevant that MyD88 ubiquitylation may take place in the context of a Myddosome oligomer that contains multiple MyD88 molecules. The positioning of the single degron described in this study may therefore be compatible with multivalent interactions with a SPOP oligomer when viewed in the context of a higher order Myddosome structure.

Many human malignancies have driver mutations in SPOP or its client proteins, and most of these directly affect recruitment by the MATH domain [45]. MyD88 is frequently mutated in Diffuse Large B-Cell Lymphoma (DLBCL), with the most prevalent mutation in this cancer, L252P (frequently referred to as L265P), occurring in the TIR domain [11]. In addition to the L252P mutation, there have been reports of other DLBCL-associated MyD88 mutations of unknown significance which occur in the SPOP-binding region [46,47]. Future studies would be required to determine if these MyD88 mutations promote DLBCL cancer progression by preventing SPOP-mediated MyD88 ubiquitylation and degradation, thereby promoting uncontrolled Myddosome signaling.

We generated a new consensus SPOP-binding degron based on the long degrons of the substrates MyD88, Pdx1, GLI2, SRC-3, SENP7, SETD2, and Caprin1. These degrons contain both the Q-motif and the established SBC. It is notable that the SBCs of the long degrons have a poor match to the classic consensus (φ-π-S-S/T-S/T) at the +4 and +5 positions. It is therefore possible that the sub-optimal SBCs are compensated by the presence of a Q-motif and that the presence of a Q-motif in a subset of SPOP substrates might lead to loss of conservation at some SBC positions during evolution. Consistent with the importance of the side chain interactions of this residue, all of the substrates we tested had glutamine at the −4 position. Although our initial search for long degrons had identified DEK as a candidate, our binding assay did not show evidence of interactions N-terminal to the SBC. The DEK degron notably has an asparagine residue at the −4 position, again consistent with the requirement of a glutamine residue at this site. The residue at the −5 position of the Q-motif is less well conserved, with a variety of residues making interactions within the hydrophobic pocket of SPOP^MATH^.

A variety of approaches have been used to systematically identify SPOP substrates, including yeast-two hybrid assays [23,27,34,42,43,48–51], ubiquitylome analysis [28,30,52], and protein interactome studies [23,53–56]. It is often challenging to identify the degrons in candidate substrates, and many substrates do not contain a classic SBC. Based on the substrates discussed in this study, and in addition to the classic SBC, we propose a new ‘long’ SPOP-binding degron, of λ-Q-X-X-X-φ-π-S-X-X (X is any amino acid, φ is a non-polar amino acid, π is a polar amino acid, and λ is a medium or large amino acid). This contains the Q-motif together with an SBC in which the +4 and +5 positions are more relaxed than the previously described consensus. E3 ligase degrons are increasingly being characterized using bioinformatics and deep learning approaches [57,58], and a major driver for such studies is the identification of new substrates and their targetable interactions that are relevant in human disease. The work described here broadens our understanding of SPOP-binding degrons and will facilitate such endeavors.

## Materials and methods

### Plasmids

All gene sequences are from *Homo sapiens*. The SPOP MATH domain (SPOP^MATH^; residues 28–116) was cloned into a modified pGEX-6P1 vector that encoded an N-terminal hexahistidine-MBP tag and an HRV 3C protease cleavage site. The full-length coding sequences of human *MYD88, SPOP, CAPRIN1, SENP7*, and *NCOA3* (*SRC-3*) were cloned into a pcDNA3.1 vector with an N-terminal 2× FLAG tag using the Thermo Scientific Fast Digest restriction enzyme system. Mutant variants were derived from these plasmids using PCR-based mutagenesis. For stable expression, MyD88 was cloned with FLAG/HA tags into the pBABE-puro (Addgene, 1764) retroviral vector, while the untagged version was cloned into the MIGR1 (Addgene, 27490) retroviral vector.

### Bacterial expression and purification of SPOP^MATH^

The SPOP^MATH^ plasmid was transformed into chemically competent *E. coli* BL21 (DE3) cells (NEB). Transformed cells were inoculated into LB medium containing 100 μg/ml ampicillin, and cultures were grown overnight at 37°C with shaking at 220 rpm. A 10 ml of overnight culture was inoculated into 1 l of Terrific Broth (Millipore) supplemented with 4 ml of glycerol and 100 μg/ml ampicillin, and cells were grown at 37°C with shaking until an optical density at 600 nm of ~0.8–1. Expression of recombinant protein was induced with 0.2 mM isopropyl-β-D-thiogalactopyranoside (IPTG), and the cells were incubated overnight at 18°C with shaking at 220 rpm. Cells were harvested by centrifugation at 4,000×g for 20 minutes, and the pellet resuspended in 60 ml lysis buffer (25 mM HEPES pH 7.4, 150 mM NaCl, 20 mM Imidazole, 0.075% β-mercaptoethanol, 1 mM Benzamidine), supplemented with 1 mM phenylmethylsulfonyl fluoride (PMSF). Cells were lysed by sonication and the lysate was clarified by centrifugation at 30,000×g for 30 minutes. Clarified lysate was incubated with 5 ml of His-Pur Ni-NTA resin (Thermo Scientific) for 1 hour, rolling, at 4°C. The Ni-NTA resin was washed with~50 ml of lysis buffer, and the protein was eluted in lysis buffer containing 200 mM Imidazole. The eluate was supplemented with ~580 μg of His-HRV 3C protease to cleave the His-MBP-tag and dialyzed overnight against 1 l of 25 mM HEPES pH 7.4, 150 mM NaCl, 1 mM dithiothreitol (DTT) using SnakeSkin 3.5 kDa cut-off dialysis membrane (Thermo Fisher). The protein was then incubated with Ni-NTA resin for 2 hours, rolling at 4°C, to remove the cleaved His-MBP tag, any uncleaved protein, and the His-tagged 3C protease. SPOP^MATH^ was further purified by size exclusion chromatography in gel filtration buffer (25 mM HEPES pH 7.4, 150 mM NaCl, 1 mM tris(2-carboxyethyl)phosphine [TCEP]) using an ÄKTA Pure system (Cytivia) with either a HiLoad 16/600 Superdex 75 pg column or a Superose6 Increase 10/300 gl column (Cytivia). Fractions containing SPOP^MATH^ were pooled and stored at −80°C.

### Fluorescence polarization (FP) assay

Peptides used for FP assays were synthesized by Peptide Synthetics (Peptide Protein Research Limited, U.K) and provided at >95% purity as assessed by HPLC (Supplementary Table 4). Fluorescently labeled peptides were labeled with 5-carboxyfluorescein (5Flu), either via the N-terminal amino group (N-terminal label) or via the side chain ε-amino group of an added C-terminal lysine (C-terminal label). Peptides and SPOP^MATH^ protein were diluted in FP buffer (50 mM HEPES pH 7.5, 100 mM NaCl, 1 mM TCEP, 0.005% Tween-20). Measurements were made in 384-well black flat-bottom low-flange plates (Corning) using 20 μl reaction volumes. Plates were equilibrated at room temperature for 30 minutes before reading. Millipolarization (mP) measurements were taken in a Hidex Sense microplate reader (Hidex) at 25°C, with excitation at 490 nm (20 nm filter), emission at 520 nm (14 nm filter), and a G-factor of 1.

For measurements of binding affinity between SPOP^MATH^ and fluorescently labeled peptides, a two-fold dilution series of SPOP^MATH^ was plated and mixed with a constant concentration of 5Flu-labeled peptide. The optimum concentration of ^5Flu^-peptide was empirically tested, with a final working concentration range between 6 and 50 nM. The technical duplicate mP values were averaged, and error bars represent the standard deviation. Data were plotted and analyzed using Prism 9 software (GraphPad Prism version 9, GraphPad Software, Boston, Massachusetts U.S.A., www.graphpad.com). Data were normalized to percent FP, where a ‘no SPOP^MATH^’ control was set to 0% FP, and the highest SPOP^MATH^ concentration tested was set to 100% FP. Dissociation constant (*K*_d_) values were calculated using a one-site total binding equation. *K*_d_ values from three independent experiments were averaged and the standard deviation was calculated.

For peptide competition measurements where unlabeled peptides competed for binding to a SPOP^MATH^–^5Flu^-peptide complex, a two-fold dilution series of unlabeled peptide was mixed with 50 nM Puc-^5Flu^ peptide (SRENLACDEVTSTTSK-^5Flu^) and 5 μM SPOP^MATH^. The concentration of SPOP^MATH^ used in this assay was determined as the concentration that produced 70% of the maximum mP signal in the binding affinity FP assay. The technical duplicate mP values were averaged, and error bars represent standard deviation. Data were plotted and analyzed using Prism 9 software. An (inhibitor) vs. response–variable slope (four parameters) curve was fit to the data to give the half maximal inhibitory concentration (IC_50_), which was calculated as the concentration of unlabeled peptide halfway between the top and the bottom of the curve. IC_50_ values from three independent experiments were averaged and the standard deviation was calculated.

### Isothermal titration calorimetry (ITC)

Experiments were performed using a Microcal™ iTC200 system (Malvern Panalytical). 300 μM of SPOP^MATH^ was titrated into 30 μM of MyD88 peptide (AEKPLQVAAVDSSVP). All samples were diluted in gel filtration buffer (25 mM HEPES pH 7.5, 150 mM NaCl, 1 mM TCEP) and degassed prior to measurements. Experiments were performed with a reference power of 5 μcalories/second, stirring speed of 750 rpm, at 25°C with a total of 20 injections. The first injection was 0.5 μL for 2 seconds, and subsequent injections were 2 μl for 4 seconds. The spacing between injections was 120 seconds. A heat of dilution control titration was performed where 300 μM SPOP^MATH^ was titrated into buffer. Results were analyzed using Origin software (Malvern). The data were normalized against the heat of dilution control, and a binding isotherm curve was fitted using a One Set of Sites model. The *K*_d_ was calculated from the reciprocal of the binding affinity (*K*a). *K*_d_s from three independent experiments were averaged and the standard deviation was calculated.

### Cell culture

HEK293T cells were maintained in Dulbecco’s modified Eagle media (Corning) containing 10% bovine serum (Gibco) and 100 U/ml penicillin, 100 μg/ml streptomycin. BJAB cells were maintained in RPMI1640 media (Gibco) containing 10% fetal bovine serum (FBS; Hyclone) and 100 U/ml penicillin, 100 μg/ml streptomycin (Gibco). Cells were incubated at 37°C with 5% CO_2_.

### Transfection and retroviral transduction

HEK293T cells were transfected using polyethylenimine (PEI; 1 mg/ml, Polysciences, #24765) dissolved in a 150 mM NaCl solution. To generate retroviruses, HEK293T cells were transfected with retroviral packaging plasmids (GP and VSVG) along with the retroviral *MYD88, CAPRIN1, SENP7*, or *NCOA3* (*SRC-3*) plasmid. The virus-containing medium was collected at 24- and 48-hour post-transfection. For viral transduction, the medium containing filtered viruses (0.45 μm membrane filter) and polybrene (2 mg/ml) was added to cells seeded at 150,000 cells/ml in six-well plates. Spin infection was carried out at 609×g for 30 minutes at room temperature. After 6 hours of incubation with the transduction reagents, the medium was replaced with fresh media. Stable cell lines were generated by selecting transduced cells with puromycin (0.5 μg/ml; Sigma-Aldrich).

### Cycloheximide-chase assay

BJAB cells stably expressing either MyD88 WT or MyD88 ^129^LQ^130^/AA were seeded in a 12-well plate at a density of 1 × 10^5^ cells per well. The cells were treated with 30 μg/ml cycloheximide (Millipore Sigma, C7698) for 0, 3, 6, and 9 hours, respectively, followed by immunoblot analysis.

### Immunoprecipitation and immunoblotting

HEK293T cells expressing FLAG-tagged proteins were treated with 5 μM MLN4924 for 6 hours before harvesting. Cells were rinsed with ice-cold phosphate-buffered saline (PBS) and lysed using NP-40 buffer (15 mM Tris, pH 7.4, 1 mM EDTA, 150 mM NaCl, 1 mM MgCl2, 10% glycerol, 0.1% NP-40) supplemented with protease inhibitors (Sigma, #11697498001). Cell lysates were incubated for 5 minutes and centrifuged at 15,000 rpm for 5 minutes at 4°C. The resulting supernatants were incubated with anti-FLAG/M2 affinity agarose beads (Sigma, A2220) for 2 hours at 4°C on a rotating platform. The immunoprecipitates were washed four times with NP-40 buffer and eluted using Laemmli buffer (240 mM Tris, pH 6.8, 8% SDS, 0.04% bromophenol blue, 5% β-mercaptoethanol, 40% glycerol). The samples were then boiled at 95°C for 5 minutes and analyzed by immunoblotting.

For immunoblot analysis, protein concentrations were measured, and samples were resolved on SDS-PAGE before being transferred to PVDF membranes (Millipore, IPVH00010). Membranes were blocked with 5% blotting-grade blocker (Bio-Rad, 1706404) in PBST (DPBS (Gibco), 0.1% Tween-20) for 30 minutes, followed by incubation with primary antibodies diluted in 5% blocker/PBST for 2 hours at room temperature. Membranes were washed with PBST and incubated overnight at 4°C with HRP-conjugated secondary antibodies. After additional washes (four times with PBST for 10 minutes and once with PBS for 15 minutes), the membranes were treated with ECL reagents (Thermo Fisher Scientific, Super Signal West Pico PLUS, 34580) and visualized using the IQ-800 system in a dark room.

Primary antibodies used included anti-MyD88 (Cell Signaling, 4283), anti-SPOP (Proteintech, 16750–1-AP), anti-FLAG (Sigma, F7425), anti-HA (Cell Signaling, 3724S), and anti-Vinculin (Santa Cruz, sc-73614). HRP-linked secondary antibodies included anti-rabbit IgG HRP (Cell Signaling, 7074S) and anti-mouse IgG HRP (GE Healthcare, NA931V). All antibodies were used at a 1:1000 dilution except anti-Vinculin, which was used at a 1:10,000 dilution.

### X-ray crystallography

For crystal plate preparation, 50–60 μl of Morpheus HT-96 protein crystallization screen (Molecular Dimensions) was added to the reservoir of an MRC 96-well two-drop crystallization plate (Molecular Dimensions). Crystals were grown at 18°C by sitting-drop vapor diffusion by mixing 200 nl of protein–peptide mixture with 200 nl of reservoir buffer using a Mosquito robot (Labtech). Crystals were picked and flash frozen in liquid nitrogen without cryoprotectant, and X-ray data were collected at the Diamond Light Source (Oxfordshire, U.K; parameters in Supplementary Table 1). Structures were solved by molecular replacement with Phenix’s Phaser-MR [59] (simple one-component interface) using a search model of apo-SPOP^MATH^ (PDB ID 7KPI) [21]. The structures underwent several rounds of iterative model building in Coot-0.9.8.1 [60] followed by refinement in Phenix-1.2.1 [61]. All peptides were built into the electron density manually. Structures were visualized in ChimeraX [62]. Omit maps were generated by setting the occupancy of the peptide to 0, followed by a round of refinement. Maps were displayed in ChimeraX [62] with the Clipper plugin (Tristan Croll, University of Cambridge, U.K) and contoured at 2.5σ.

For the MyD88 peptide (^125^AEKPLQVAAVDSSVPRT^141^)-SPOP^MATH^ structure (PDB ID: 9HGH), peptide (1.75 mM final concentration) was mixed with SPOP^MATH^ (552 μM final concentration), in buffer containing 25 mM HEPES pH 7.4, 150 mM NaCl, 1 mM TCEP and incubated on ice for 4 hours before mixing with precipitant. Data were collected on a crystal picked after 11 days from a Morpheus HT-96 crystal screen (well condition E12; 12.5% w/v Peg 1000, 12.5% w/v Peg 3350, 12.5% v/v MPD, 0.03 M diethyleneglycol, 0.03 M triethyleneglycol, 0.03 M tetraethyleneglycol, 0.03 M pentaethyleneglycol, 0.1 M bicine/Trizma base pH 8.5), which diffracted to 1.7 Å, later truncated to 1.9 Å (Supplementary Table 1). Data were processed using xia2-3dii [63].

For the MyD88 peptide (^127^KPLQVAAVDSSVPRTAELAG^146^)-SPOP^MATH^ structure (PDB ID: 9HFV), peptide (1.75 mM final concentration) was mixed with SPOP^MATH^ (552 μM final concentration), in buffer containing 25 mM HEPES pH 7.4, 150 mM NaCl, 0.5 mM TCEP and incubated on ice for 4 hours before mixing with precipitant. Data were collected on a crystal picked after 18 days from a Morpheus HT-96 crystal screen (well condition A5; 10% w/v Peg 20,000, 20% v/v Peg MME 550, 0.03 M magnesium chloride, 0.03 M calcium chloride, 0.1 M MOPS/HEPES-Na pH 7.5) which diffracted to 1.2 Å, later truncated to 1.45 Å (Supplementary Table 1). Data were processed using xia2-3dii [63].

For the Caprin1 peptide (^426^QPEATQVPLVSSTSEGY^442^)-SPOP^MATH^ structure (PDB ID: 9HFU), peptide (1.66 mM final concentration) was mixed with SPOP^MATH^ (552 μM final concentration), in buffer containing 25 mM HEPES pH 7.4, 150 mM NaCl, 1 mM TCEP and incubated on ice for 2 hours before mixing with precipitant. Data were collected on a crystal picked after 17 days from a Morpheus HT-96 crystal screen (well condition B12; 12.5% w/v Peg 1000, 12.5% w/v Peg 3350, 12.5% v/v MPD, 0.03 M sodium fluoride, 0.03 M sodium bromide, 0.03 M sodium iodide, 0.1 M bicine/Trizma base pH 8.5) which diffracted to 1.48 Å, later truncated to 1.7 Å (Supplementary Table 1). Data were processed using autoPROC [64].

For the SRC-3 peptide (^91^NDDDVQKADVSSTGQGV^107^)-SPOP^MATH^ structure (PD B ID:9HFW), peptide (1.6 mM final concentration) was mixed with SPOP^MATH^ (499 μM final concentration), in buffer containing 25 mM HEPES pH 7.4, 150 mM NaCl, 1 mM TCEP and incubated on ice for 2 hours before mixing with precipitant. Data were collected on a crystal picked after seven days from a Morpheus HT-96 crystal screen (well condition F4; 12.5% w/v Peg 1000, 12.5% w/v Peg 3350, 12.5% v/v MPD, 0.03 M sodium fluoride, 0.03 M sodium bromide, 0.03 M sodium iodide, 0.1 M bicine/Trizma base pH 8.5) which diffracted to 1.52 Å, later truncated to 1.7 Å (Supplementary Table 1). Data were processed using autoPROC [64].

For the SETD2 peptide (^363^DKGSVQAPEISSNSIKD^1379^)-SPOP^MATH^ structure (PDB ID: 9HGG), peptide (2.6 mM final concentration) was mixed with SPOP^MATH^ (880 μM final concentration), in buffer containing 25 mM HEPES pH 7.4, 150 mM NaCl, 1 mM TCEP and incubated on ice for 2 hours before mixing with precipitant. Data were collected on a crystal picked after 11 days from a Morpheus HT-96 crystal screen (well condition A1; 10% w/v Peg 20 000, 20% v/v Peg MME 550, 0.03 M sodium fluoride, 0.03 M sodium bromide, 0.1 M MES/imidazole pH 6.5) which diffracted to 1.57 Å, later truncated to 1.7 Å (Supplementary Table 1). Data were processed using xia2 dials [63].

## Supplementary Material

Supplementary Material

## Data Availability

X-ray structure factors and associated co-ordinates have been deposited at the PDB under accession codes 9HGH (MyD88_1), 9HFV (MyD88_2), 9HFU (Caprin1), 9HFW (SRC-3), and 9HGG (SETD2).

## Abbreviations

FP: fluorescence polarization
ITC: isothermal titration calorimetry
SBC: SPOP-binding consensus
SPOP: speckle-type BTB/POZ protein
SRC-3: steroid receptor coactivator-3
TLRs: toll-like receptors
