# Supplementary material for: Sequence rules for a long SPOP-binding degron required for protein ubiquitylation
Source: Biochem J. Author manuscript; Available in PMC 2025 Oct 6. (PMC7618200; doi:10.1042/BCJ20253041)
Supplement: Supplementary Material [file EMS209254-suppement-Supplementary_Material.pdf]

References from supplementary materials

69 Crooks, G.E., Hon, G., Chandonia, J.-M. and Brenner, S.E. (2004) WebLogo: a sequence logo generator. *Genome Res.* **14**, 1188–1190 https://doi.org/10.1101/gr.849004

70 Wu, F., Dai, X., Gan, W., Wan, L., Li, M., Mitsiades, N. et al. (2017) Prostate cancer-associated mutation in SPOP impairs its ability to target Cdc20 for poly-ubiquitination and degradation. *Cancer Lett.* **385**, 207–214 https://doi.org/10.1016/j.canlet.2016.10.021

71 Luo, J., Bao, Y., Ji, X., Chen, B., Deng, Q. and Zhou, S. (2017) SPOP promotes SIRT2 degradation and suppresses non-small cell lung cancer cell growth. *Biochem. Biophys. Res. Commun.* **483**, 880–884 https://doi.org/10.1016/j.bbrc.2017.01.027

© 2025 The Author(s). This is an open access article published by Portland Press Limited on behalf of the Biochemical Society and distributed under the Creative Commons Attribution License 4.0 (CC BY).

72 Zhang, L., Peng, S., Dai, X., Gan, W., Nie, X., Wei, W. et al. (2017) Tumor suppressor SPOP ubiquitinates and degrades EglN2 to compromise growth of prostate cancer cells. *Cancer Lett.* **390**, 11–20 https://doi.org/10.1016/j.canlet.2017.01.003

73 Geng, C., Kaochar, S., Li, M., Rajapakshe, K., Fiskus, W., Dong, J. et al. (2017) SPOP regulates prostate epithelial cell proliferation and promotes ubiquitination and turnover of c-MYC oncoprotein. *Oncogene* **36**, 4767–4777 https://doi.org/10.1038/onc.2017.80

74 Tan, Y., Ci, Y., Dai, X., Wu, F., Guo, J., Liu, D. et al. (2017) Cullin 3SPOP ubiquitin E3 ligase promotes the poly-ubiquitination and degradation of HDAC6. *Oncotarget* **8**, 47890–47901 https://doi.org/10.18632/oncotarget.18141

75 Gang, X., Xuan, L., Zhao, X., Lv, Y., Li, F., Wang, Y. et al. (2019) Speckle-type POZ protein suppresses lipid accumulation and prostate cancer growth by stabilizing fatty acid synthase. *Prostate* **79**, 864–871 https://doi.org/10.1002/pros.23793

76 Jiang, Q., Zheng, N., Bu, L., Zhang, X., Zhang, X., Wu, Y. et al. (2021) SPOP-mediated ubiquitination and degradation of PDK1 suppresses AKT kinase activity and oncogenic functions. *Mol. Cancer* **20** https://doi.org/10.1186/s12943-021-01397-5

77 Su, S., Chen, J., Jiang, Y., Wang, Y., Vital, T., Zhang, J. et al. (2021) SPOP and OTUD7A Control EWS-FLI1 Protein stability to govern ewing sarcoma growth. *Adv. Sci.***8**, 2004846 https://doi.org/10.1002/advs.202004846

78 Feng, K., Shi, Q., Jiao, D., Chen, Y., Yang, W., Su, K. et al. (2022) SPOP inhibits BRAF-dependent tumorigenesis through promoting non-degradative ubiquitination of BRAF. *Cell Biosci.* **12**, 211–211. https://doi.org/10.1186/s13578-022-00950-z

79 Schwartz, I., Vunjak, M., Budroni, V., Cantoran García, A., Mastrovito, M., Soderholm, A. et al. (2023) SPOP targets the immune transcription factor IRF1 for proteasomal degradation. *Elife* **12**, e89951 https://doi.org/10.7554/eLife.89951

80 Krissinel, E. and Henrick, K. (2007) Inference of macromolecular assemblies from crystalline state. *J. Mol. Biol.* **372**, 774–797 https://doi.org/10.1016/j.jmb.2007.05.022

© 2025 The Author(s). This is an open access article published by Portland Press Limited on behalf of the Biochemical Society and distributed under the Creative Commons Attribution License 4.0 (CC BY).

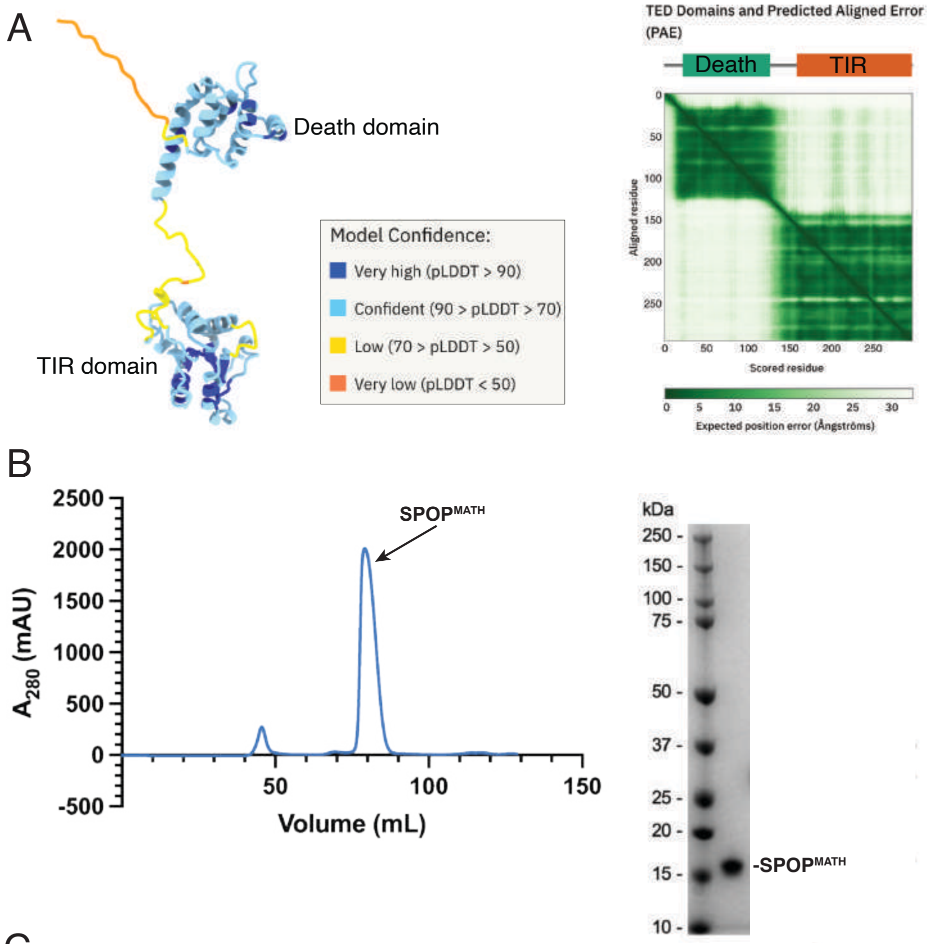

C

N (sites)	0.82 ±0.06
Ka (M^{-1})	1.94E6 ±4.99E5
ΔH (cal mol^{-1})	-1.04E4 ±3.13E2
ΔS (cal mol^{-1} $^{\circ -1}$)	-6.11 ±0.73

Supplementary Figure 1 – The SPOP-interacting region of MyD88

A Left - AlphaFold2 [(66-68)] model of MyD88 coloured by predicted local distance difference test (pLDDT). Right – associated TED domains and predicted alignment error (PAE) plot.

B Purification of the $SPOP^{MATH}$ domain, showing the size-exclusion chromatography trace together with an SDS-PAGE gel of the purified protein.

C ITC parameters for the titration of $SPOP^{MATH}$ into a MyD88 peptide (see **Figure 1E**).

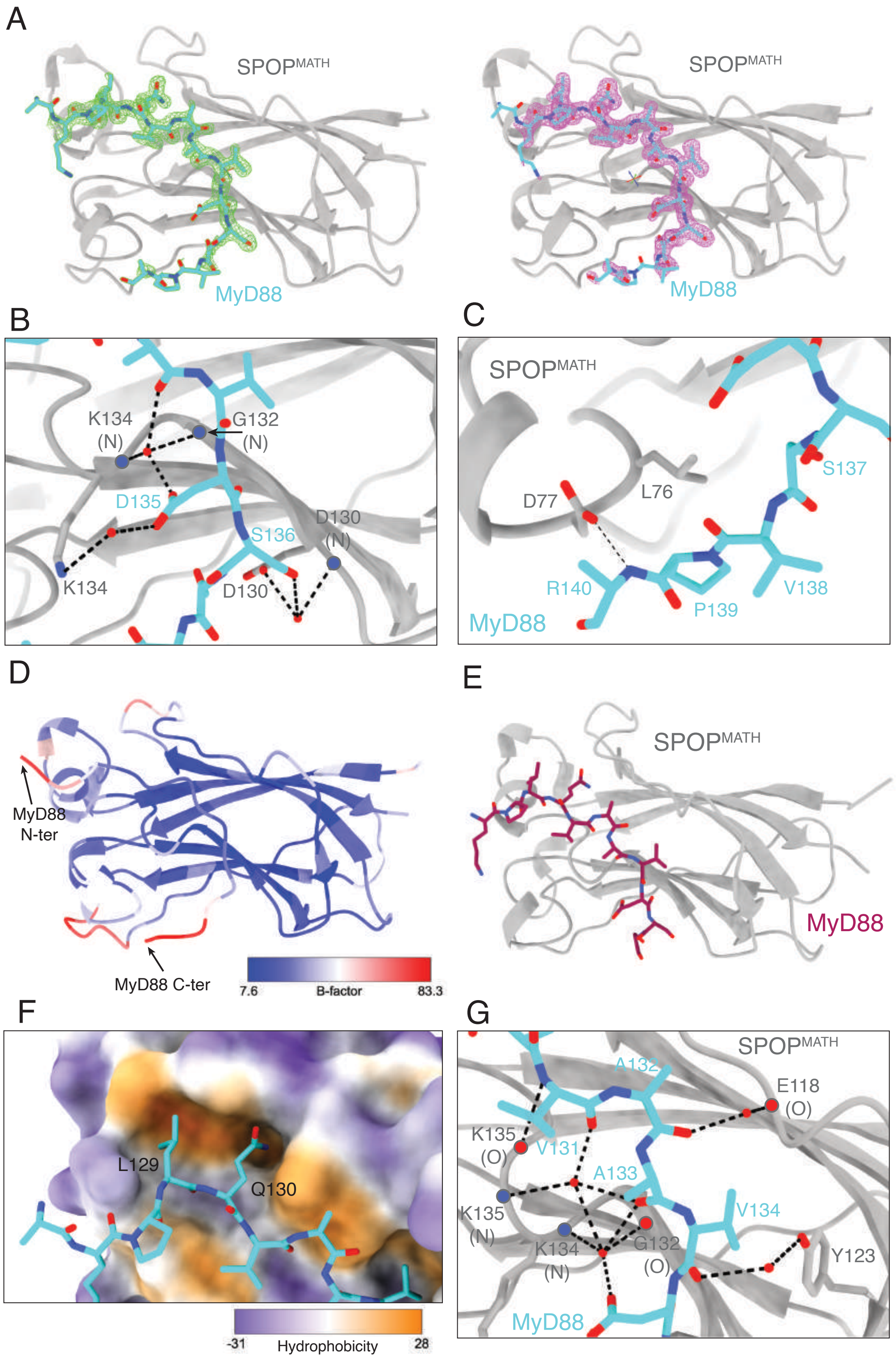

Supplementary Figure 2 – Structural basis of the SPOP^{MATH}-MyD88 interaction

A Left - Omit map (Fo-Fc) of the MyD88 ^{125}AEKPLQVAAVDSSVPRT^{141} peptide contoured at 2.5σ. Right – Final peptide 2Fo-Fc density contoured at 1σ (see **Figure 2A**).

B Detailed view of water-mediated contacts between SPOP^{MATH} and MyD88 ^{135}DSS^{137} (see **Figure 2B**).

C Detailed view of interactions between SPOP^{MATH} and the C-terminus of the MyD88 peptide (see **Figure 2A**).

D B-factor analysis of the MyD88 ^{125}AEKPLQVAAVDSSVPRT^{141} peptide (see **Figure 2A**).

E Crystal structure of SPOP^{MATH} in complex with a MyD88 peptide (^{127}KPLQVAAVDSSVPRTAELAG^{146}). SPOP^{MATH} is displayed as a cartoon representation in grey, and the MyD88 peptide is shown in stick representation in purple. MyD88 residues ^{138}VPRTAELAG^{146} were not built into the model.

F MyD88 residues Leu^{129} and Gln^{130} are located in a hydrophobic pocket of SPOP^{MATH} (see **Figure 2B**).

G Detailed view of water-mediated contacts between SPOP^{MATH} and MyD88 ^{131}VAA^{133} (see **Figure 2A**).

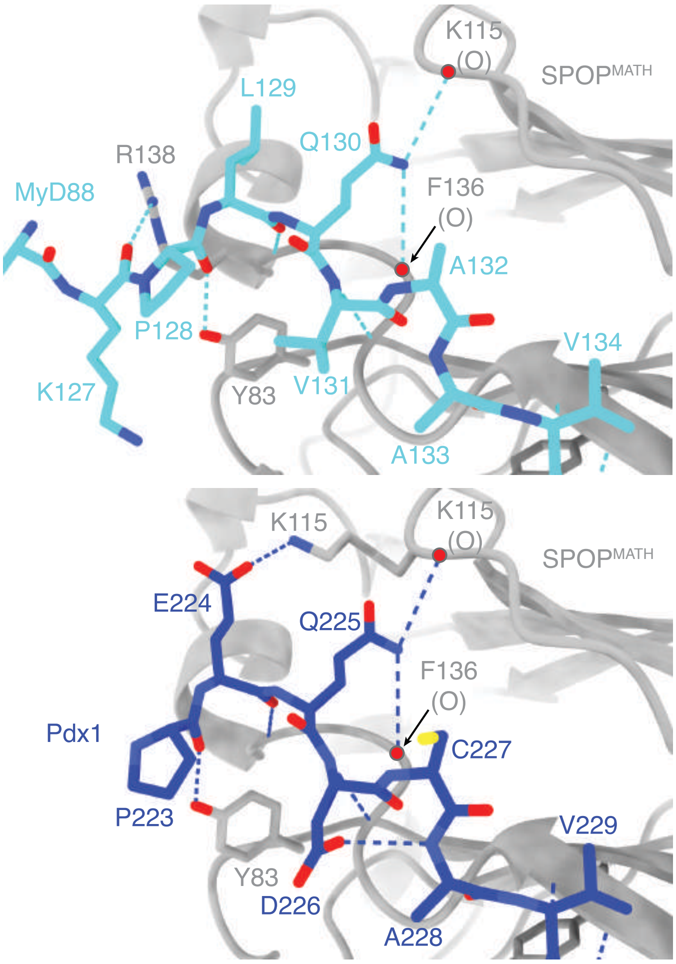

Supplementary Figure 3 – SPOP substrate degrons – importance of both the SBC and an N-terminal Q motif

Interaction of SPOP^{MATH} with peptides from the substrates MyD88 and Pdx1 (PDB ID: 6F8F) [20]. H-bonds are shown as dashed lines.

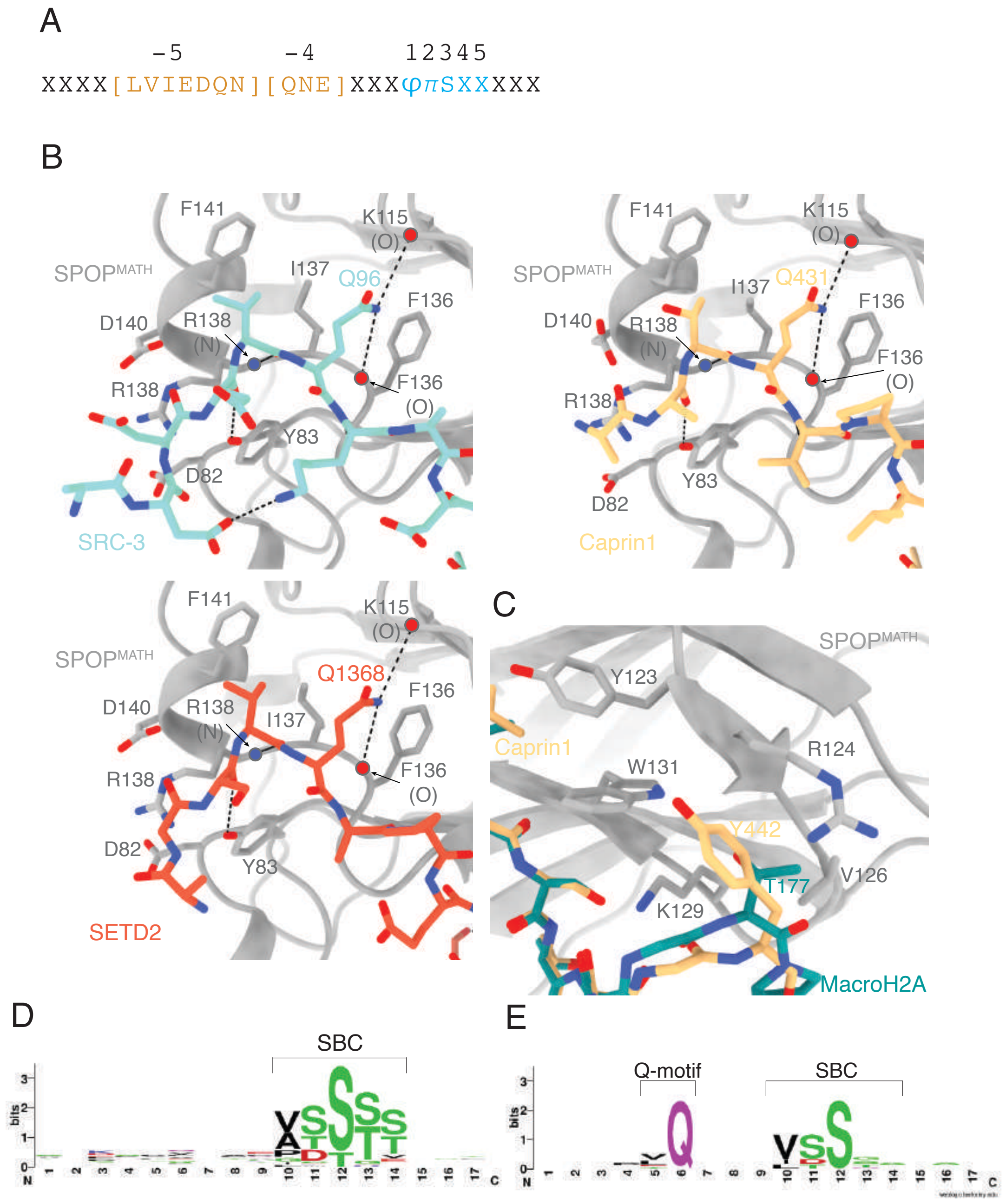

Supplementary Figure 4 - Additional substrates interact with SPOP^{MATH} via a Q-motif

A Sequence of the search motif used to search for additional substrates that interact with SPOP^{MATH} via a Q-motif. X represents any amino acid. ϕ represents a non-polar amino acid and π represents a polar amino acid.

B Detailed views of interactions between SPOP^{MATH} and the Q-motif region of the SRC-3, SETD2 and Caprin1 peptides (see **Figure 5E**).

C Structural superposition of $SPOP^{MATH}$-Caprin1 and $SPOP^{MATH}$-MacroH2A (PDB ID: 3IVB) [(2)]. Superposition was by structural alignment of the MATH domains. $SPOP^{MATH}$ from the complex with Caprin1 is displayed as a cartoon in grey. Caprin1 peptide is in yellow and the MacroH2A peptide in teal.

D The SPOP-binding consensus (SBC). The SBC was defined using sequences from known SPOP substrates (**Supplementary Table 2)**. The motif search was carried out using WebLogo software (University of California, Berkeley, USA) [(69)]. Green residues are polar. Black residues are hydrophobic. Purple residues are neutral. Red residues are acidic. Blue residues are basic.

E Expanded SPOP-binding consensus motif derived from the sequences of the Q-motif containing substrates MyD88, Pdx1, GLI2, SRC-3, SENP7, SETD2 and Caprin1.

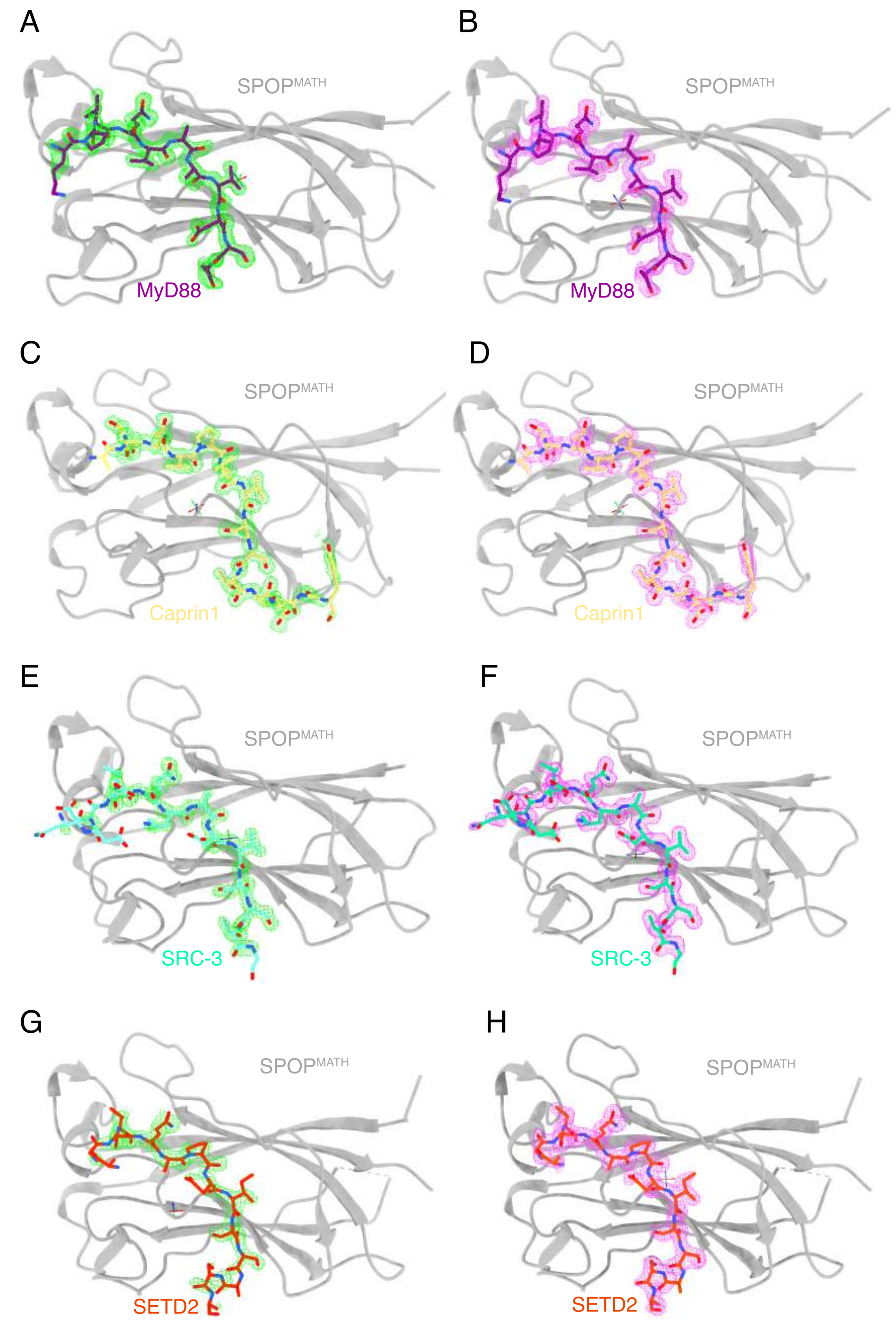

Supplementary Figure 5 – Omit and final peptide maps

A Omit map (Fo-Fc) of the MyD88 ^{127}KPLQVAAVDSSVPRTAELAG^{146} peptide contoured at 2.5σ.

B Final peptide 2*F*o-*F*c density contoured at 1σ as in (**A**).

C Omit map (*F*o-*F*c) of the Caprin1 ^{426}QPEATQVPLVSSTSEGY^{442} peptide contoured at 2.5σ.

D Final peptide 2*F*o-*F*c density contoured at 1σ as in (**C**).

E Omit map (*F*o-*F*c) of the SRC-3 ^{91}NDDDVQKADVSSTGQGV^{107} peptide contoured at 2.5σ.

F Final peptide 2*F*o-*F*c density contoured at 1σ as in (**E**).

G Omit map (*F*o-*F*c) of the SETD2 ^{363}DKGSVQAPEISSNSIKD^{1379} peptide contoured at 2.5σ.

H Final peptide 2*F*o-*F*c density contoured at 1σ as in (**G**).

	MyD88_1	Caprin1	SRC-3	SETD2	MyD88_2
**Peptide sequence:**	[125]AEKPLQVAA VDSSVPRT[141]	[426]QPEATQVP LVSSTSEGY[442]	[91]NDDDVQKAD VSSTGQGV[107]	[1363]DKGSVQA PEISSNSIKD[1379]	[127]KPLQVAAVDS SVPRTAELAG[146]
**Data collection:**					
**Beam line**	I24 microfocus			I04 microfocus	I24 microfocus
**Wavelength (Å)**	0.99987	0.6199	0.6199	0.9537	0.6199
**Space group**	$P2_12_12_1$	$P12_11$	$P2_12_12$	$P22_12_1$	$P2_12_12_1$
**Cell dimensions**					
**a, b, c (Å)**	40.00, 57.29, 60.97	39.81, 60.88, 57.12	53.04, 54.28, 45.88	44.56, 51.04, 54.90	39.83, 57.08, 61.24
**α, β, γ (°)**	90.00, 90.00, 90.00	90.00, 90.05, 90.00	90.00, 90.00, 90.00	90.00, 90.00, 90.00	90.00, 90.00, 90.00
**Resolution**	33.44-1.90 (1.97-1.90)	41.67-1.70 (1.76-1.70)	45.88-1.70 (1.76-1.70)	44.56-1.90 (1.97-1.90)	41.77-1.45 (1.50-1.45)
**Total reflections**	66086	57086	29829	132443	332266
**Unique reflections**	11453 (1091)	29981 (2918)	15071 (1420)	10343 (1018)	25391 (2472)
**Completeness (%)**	99.4 (97.8)	99.5 (98.7)	99.7 (97.1)	100.0 (100.0)	99.9 (98.8)
**R_{merge} (%)**	19.2 (85.5)	6.7 (26.1)	3.1 (19.2)	18.4 (67.1)	7.1 (70.0)
**$R_{p.i.m.}$ (%)**	8.7 (41.9)	6.7 (26.1)	3.1 (19.2)	5.4 (18.9)	2.1 (21.4)
**I/σI**	9.8 (4.9)	6.9 (3.3)	11.6 (4.7)	45.9 (1.0)	20.5 (3.9)
**Multiplicity**	5.8 (4.9)	1.9 (1.8)	2.0 (1.8)	12.8 (13.6)	13.1 (11.1)
**$CC_{1/2}$**	0.99 (0.76)	0.99 (0.85)	1.0 (0.80)	0.99 (0.85)	1.0 (0.88)
**Refinement:**					
**R_{work}/R_{free}**	0.1730/0.2322	0.1624/0.2039	0.1779/0.2233	0.1942/0.2561	0.1631/0.2138
**B-factors (Å²)**	22.50	19.07	18.72	32.15	20.88
**RMS bonds (Å)**	0.011	0.011	0.012	0.012	0.010
**RMS angles (°)**	1.132	1.027	1.124	1.212	1.030
**Ramachandran favored (%)**	97.33	96.70	97.93	97.26	97.97
**Ramachandran outliers (%)**	0.67	0.00	0.00	0.00	0.00
**Number of water molecules**	121	247	132	121	169

Supplementary Table 1 – X-ray crystallography data collection and refinement statistics of SPOP^{MATH}-peptide crystal structures

Highest resolution shell information is in parentheses. R_{free} was calculated using a random 5% of data that was not used for refinement. Models were refined using Phenix refine and statistics were calculated using MolProbity.

Protein	SBC region sequence	UniProt ID	Reference
**MyD88_1**	^{5}GPGA**GS**AAPVSSTSSLP^{21}	Q99836	(16)
**MyD88_2**	^{125}AEKP**LQ**VAAVDSSVPRT^{141}	"	"
**Pdx1**	^{220}VAEP**EQ**DCAVTSGEELL^{236}	P52945	(21)
**Puc_1**	^{89}SREN**LA**CDEVTSTTSSS^{105}	O46122	(2)
**Puc_2**	^{280}TPHL**NS**PSNPSSSSVGL^{296}	"	"
**Puc_3**	^{372}ASSV**SE**LDSPSSTSSSS^{388}	"	"
**CI_1**	^{362}PFKD**VV**PEQPSSTSGGV^{378}	P19538	(27)
**CI_2**	^{1353}PVNN**TL**FPDVSSSTHPY^{1369}	"	"
**GEMININ**	^{190}EIGT**CA**EGTVSSSTDAK^{206}	O75496	(23)
**TP53BP1**	^{1632}NVSS**PA**TPTASSSSSTT^{1648}	Q12888	(24)
**BRD3**	^{237}VKKK**GV**KRKADTTTPTT^{253}	Q15059	(28)
**MacroH2A**	^{162}QGEV**SK**AASADSTTEGT^{178}	O75367	(2)
**DAXX_1**	^{505}EKNL**EP**GKQISRSSGEQ^{521}	Q9UER7	(29)
**DAXX_2**	^{578}TPSS**VV**ETDISSSRKQS^{593}	"	(29)
**DAXX_3**	^{599}TVLE**NG**AGMVSSTSFNG^{615}	"	(2, 29)
**DAXX_4**	^{671}SPLA**SL**APVADSSTRVD^{687}	"	(2, 29)
**DAXX_5**	^{685}RVDS**PS**HGLVTSSLCIP^{701}	"	(29)
**GLI2_1**	^{1140}NNMP**VQ**WNEVSSGTVDA^{1156}	P10070	(41)
**GLI2_2 (?)**	^{353}QNKQ**SS**ESAVSSTVNPV^{369}	"	
**GLI2_3 (?)**	^{621}EPGG**PE**STEASSTSQAV^{637}	"	
**GLI2_4 (?)**	^{1487}PLPS**PG**VNQVSSTVDSQ^{1503}	"	
**GLI3_1**	^{1478}ELLS**PG**ANQVTSTVDSL^{1494}	P10071	(7)
**GLI3_2**	^{26}RTDV**SE**KAVASSTTSNE^{42}	"	"
**GLI3_3**	^{67}QGLS**KV**SEEPSTSSDER^{82}	"	"
**DEK**	^{276}SVKS**AN**VKKADSSTTKK^{292}	P35659	(30)
**BRD4**	^{283}KTKK**GV**KRKADTTTPTT^{299}	O60885	(48)
**SRC-3/NCoA-3**	^{91}NDDD**VQ**KADVSSTGQGV^{107}	Q9Y6Q9	(42)
**DDIT3**	^{64}TEEE**PE**PAEVTSTSQSP^{80}	P35638	(31)
**ERG_1**	^{26}PHLA**KT**EMTASSSSDYG^{42}	P11308	(32, 44)
**ERG_2**	^{431}VAPH**PP**ALPVTSSSFFA^{447}	"	(32, 44)
**SENP7_1**	^{192}DTDN**LQ**SEQLSSSSDGS^{208}	Q9BQF6	(33)
**SENP7_2**	^{378}LSNA**TK**SASAGSTTETV^{394}	"	"
**SETD2**	^{1363}DKGS**VQ**APEISSNSIKD^{1379}	Q9BYW2	(34)
**CDC20**	^{52}AGRT**PG**RTPGKSSSKVQ^{68}	Q12834	(70)
**SIRT2 (?)**	^{78}RVIC**LV**GAGISTSAGIP^{94}	Q8IXJ6	(71)
**EGLN2_1**	^{10}LSQA**LP**QLPGSSSEPLE^{26}	Q96KS0	(72)
**EGLN2_2**	^{59}AGSG**TP**RATATSTTASP^{75}	"	"
**C-MYC_1**	^{176}SPNP**AR**GHSVCSTSSLY^{192}	P01106	(73)
**C-MYC_2**	^{252}PLVL**HE**ETPPTTSSDSE^{268}	"	"
**INF2**	^{1136}SLLG**VL**QAEADSTSEGL^{1152}	Q27J81	(49)
**HDAC6**	^{836}VMKV**ED**REGPSSSKLVT^{852}	Q9UBN7	(74)
**AR**	^{637}NLKL**QE**EGEASSTTSPT^{653}	P10275	(35)
**ERa**	^{563}TDQS**HL**ATAGSTSSHSL^{579}	P03372	(36)
**HIPK2_1**	^{89}STGH**IV**VTSASSTSVTG^{105}	Q9H2X6	(37)

**HIPK2_2**	^{854}SVTC**GW**GDVASSTTRER^{870}	“	“
**BRD2**	^{274}AKKK**GV**KRKADTTTPTP^{290}	P25440	(48)
**NANOG**	^{57}SMDL**LI**QDSPDSSTSPK^{73}	Q9H9S0	(38)
**ATF2_1**	^{183}VIIQ**QA**VPSPTSSTVIT^{199}	P15336	(50)
**ATF2_2**	^{310}SRPQ**SL**QQPATSTTETP^{326}	“	“
**FASN_1**	^{151}RGPS**IA**LDTACSSSLMA^{167}	A0A0U1RQF0	(75)
**FASN_2**	^{1704}AYLQ**AR**FPQLDSTSFAN^{1720}	“	“
**Caprin1**	^{426}QPEA**TQ**VPLVSSTSEGY^{442}	Q14444	(43)
**CDCA5**	^{113}TSTP**VP**NPEAESSSKEG^{129}	Q96FF9	(53)
**PDPK1**	^{379}LLSQ**FG**CMQVSSSSSSH^{395}	O15530	(76)
**DHX9**	^{332}WSPP**QS**NWNPWTSSNID^{348}	Q08211	(52)
**BRMS1**	^{180}WWDD**KL**HARGSSRSWDS^{196}	Q9HCU9	(39)
**FLI-1**	^{401}VPPH**PS**SMPVTSSSFFG^{417}	Q01543	(77)
**PTEN**	^{350}TVEE**PS**NPEASSSTSVT^{365}	P60484	(26)
**BRAF**	^{111}VSSS**AS**MDTVTSSSSSS^{127}	P15056	(78)
**GLP_1**	^{636}KAKE**VT**IAKADTTSTVT^{652}	Q9H9B1	(51)
**GLP_2**	^{658}EKGS**AL**EGRADTTTGSA^{674}	"	“
**IRF1**	^{143}SSPD**TF**SDGLSSSTLPD^{159}	P10914	(79)
**IRF2BP2**	^{438}SHAS**KD**ANQVHSTTRRN^{454}	Q7Z5L9	(54)
**ZBTB3_1**	^{187}NSQL**PS**LEFLSSTSRGT^{203}	Q9H5J0	(56)
**ZBTB3_2**	^{263}PVAD**VS**LASPSSSTETI^{279}	“	“

Supplementary Table 2 - List of SPOP substrates used for the search for long SPOP degrons

SBC is SPOP binding consensus sequence. SBC sequence underlined. Q-motif region is in bold. Proteins with more than one SBC are numbered (i.e., _1). Proteins with a (?) are known SPOP binders but with an unknown SBC sequence. A predicted SBC based on sequence similarity is suggested.

Protein	Peptide sequence	N-ter label	C-ter label	Mutation
MyD88	^{133}AVDSSVP^{139}	5Flu	-	-
MyD88	^{125}AEKPLQVAAVDSSVP^{139}	5Flu	-	-
MyD88	^{133}AVDSSVP^{139}	-	-	-
MyD88	^{133}AVDSSVPRT^{141}	-	-	-
MyD88	^{125}AEKPLQVAAVDSSVPRT^{141}	-	-	-
MyD88	^{125}AEKPLQVAAVDSSVP^{139}	-	-	-
MyD88	^{128}PLQVAAVDSSVPRT^{141}	-	-	-
MyD88	^{128}PLQVAAVDSSVP^{139}	-	-	-
MyD88	^{125}AEKPLQVAAVAAAVPRT^{141}	-	-	^{135}DSS^{137} to AAA
MyD88	^{133}AVAAAVP^{139}	-	-	^{135}DSS^{137} to AAA
MyD88	^{125}AAKPLQVAAVDSSVPRT^{141}	-	-	E126A
MyD88	^{125}AEAPLQVAAVDSSVPRT^{141}	-	-	K127A
MyD88	^{125}AEKPAQVAAVDSSVPRT^{141}	-	-	L129A
MyD88	^{125}AEKPLAVAAVDSSVPRT^{141}	-	-	Q130A
MyD88	^{125}AEKPLQVAARDSSVPRT^{141}	-	-	V134R
MyD88	^{125}AEKPLQVAAVASSVPRT^{141}	-	-	D135A
MyD88	^{125}AEKPLQVAAVDASVPRT^{141}	-	-	S136A
MyD88	^{125}AEKPLQVAAVDSAVPRT^{141}	-	-	S137A
Pdx1	^{220}VAEPEQDCAVTSGEE^{234}	5Flu	-	-
Puc	^{90}SRENLACDEVTSTTS^{104}	5Flu	-	-
Puc	^{90}SRENLACDEVTSTTS^{104}	-	K-5Flu	-
Puc	^{90}SRENLACDEVTSTTS^{104}	-	-	-
MyD88-Puc chimera	AEKPLQVAAVTSTTS	-	-	-
MyD88-Puc chimera	AEKPLAVAAVTSTTS	-	-	Q to A
Gli2	^{1140}NNMPVQWNEVSSGTVD^{1156}	-	-	-
Gli2	^{1149}EVSSGTVD^{1156}	-	-	-
DEK	^{276}SVKSANVKKADSSTTKK^{292}	-	-	-
DEK	^{284}KADSSTTKK^{292}	-	-	-
SRC3	^{91}NDDDVQKADVSSTGQGV^{107}	-	-	-
SRC3	^{99}DVSSTGQGV^{107}	-	-	-
SENP7	^{192}DTDNLQSEQLSSSSDGS^{208}	-	-	-
SENP7	^{200}QLSSSSDGS^{208}	-	-	-
SETD2	^{1363}DKGSVQAPEISSNSIKD^{1379}	-	-	-
SETD2	^{1371}EISSNSIKD^{1379}	-	-	-
CAPRIN1	^{426}QPEATQVPLVSSTSEGY^{442}	-	-	-
CAPRIN1	^{434}LVSSTSEGY^{442}	-	-	-

Supplementary Table 4 – Peptides used in fluorescence polarization assays

Residue numbers are indicated. MyD88-Puc is a chimera peptide with ^{125}AEKPLQVAA^{133} from MyD88 and ^{99}VTSTTS^{104} from Puc. 5Flu is 5-carboxyfluorescein. N-ter is N-terminus. C-ter is C-terminus.
